# Ultralow-frequency neural entrainment to pain

**DOI:** 10.1371/journal.pbio.3000491

**Published:** 2020-04-13

**Authors:** Yifei Guo, Rory John Bufacchi, Giacomo Novembre, Marina Kilintari, Massieh Moayedi, Li Hu, Gian Domenico Iannetti

**Affiliations:** 1 Neuroscience and Behaviour Laboratory, Istituto Italiano di Tecnologia, Rome, Italy; 2 Department of Neuroscience, Physiology and Pharmacology, University College London, London, United Kingdom; 3 Faculty of Dentistry, University of Toronto, Toronto, Canada; 4 CAS Key Laboratory of Mental Health, Institute of Psychology, Chinese Academy of Sciences, Beijing, China; 5 Department of Psychology, University of Chinese Academy of Sciences, Beijing, China; New York University, UNITED STATES

## Abstract

Nervous systems exploit regularities in the sensory environment to predict sensory input, adjust behavior, and thereby maximize fitness. Entrainment of neural oscillations allows retaining temporal regularities of sensory information, a prerequisite for prediction. Entrainment has been extensively described at the frequencies of periodic inputs most commonly present in visual and auditory landscapes (e.g., >0.5 Hz). An open question is whether neural entrainment also occurs for regularities at much longer timescales. Here, we exploited the fact that the temporal dynamics of thermal stimuli in natural environment can unfold very slowly. We show that ultralow-frequency neural oscillations preserved a long-lasting trace of sensory information through neural entrainment to periodic thermo-nociceptive input as low as 0.1 Hz. Importantly, revealing the functional significance of this phenomenon, both power and phase of the entrainment predicted individual pain sensitivity. In contrast, periodic auditory input at the same ultralow frequency did not entrain ultralow-frequency oscillations. These results demonstrate that a functionally significant neural entrainment can occur at temporal scales far longer than those commonly explored. The non-supramodal nature of our results suggests that ultralow-frequency entrainment might be tuned to the temporal scale of the statistical regularities characteristic of different sensory modalities.

## Introduction

The sensory environment is dynamic in nature, with its temporal structures unfolding across multiple timescales. Time is therefore an indispensable aspect of sensory experiences. The ability of the brain to track and predict the temporal dynamics of sensory inputs allows an organism to take appropriate actions to meet the changing environmental demands. What neural processes may be responsible for these functions, however, is still an open question. Accumulating evidence supports a theory that neural codes of temporal information build on brain oscillations [1–3]. Taking situations involving rhythmic sensory inputs as an example, the brain may adapt to the external rhythm through entrainment of ongoing neural oscillations at the corresponding frequency [2,4–6]. Thus, neural entrainment constitutes a flexible mechanism through which the brain adjusts the power or the phase of ongoing oscillations as a function of sensory input, with consequences on brain dynamics that can persist after the sensory input has ceased [7,8].

Temporal dynamics are ubiquitous in sensory domains, including pain [9–13]. Still, most neuroimaging studies investigating the neural mechanisms of pain were conducted using transient painful stimulation [14–16]. This approach poses two main problems. First, the neural responses to transient painful stimulation are dominated by supramodal neural activities—i.e., activities associated with the detection of salient environmental events regardless of their modality [17–19]—which limits the usefulness of this approach in identifying nociceptive-specific brain processing [16]. Second, the presentation of a single intense painful stimulus does not capture the rich and often long-lasting dynamics of pain perception, leaving the critical question of how the brain processes dynamic pain information largely unanswered. There is, therefore, a growing consensus in the field that a shift is needed from measuring brain responses elicited by transient painful stimulation to more naturalistic approaches that allow the capture of the temporal dynamics of pain [15,16].

Some attempts have been made in this new direction [10,20–25]. Tonic and fluctuating nociceptive stimuli delivered at temporal scales of seconds to minutes have been used to better simulate the dynamics of spontaneous pain in clinical conditions [14,15]. A small number of studies tried to relate the temporal profile of brain activity sampled using electroencephalography (EEG) to that of either nociceptive input or reported pain. The main observations were a relationship between the power of alpha (8–12 Hz) and beta (13–30 Hz) oscillations in sensorimotor areas and the fluctuations of nociceptive input [21,22,25], as well as between the power of gamma oscillations (>30 Hz) in medial prefrontal cortex and fluctuations of pain intensity [22,25]. In other words, the slow temporal dynamics of both nociceptive input and pain seem to be reflected in neural oscillations at frequencies several orders of magnitude higher than that of ongoing pain.

The above considerations lead to an outstanding question about the role of low-frequency neural oscillations: can the slow dynamics of pain be represented in neural oscillations at the same timescales? This is physiologically plausible, for several reasons. First, a 1:1 phase locking between the rhythm of external sensory inputs and neural oscillations is theoretically possible [26–28] and has been, in fact, repeatedly observed in auditory and visual domains in the form of neural entrainment [7,29–33]. In these cases, the neural oscillations, and specifically their phase structures, may serve as a substrate of temporal representation and prediction of the incoming input [1,2]. Second, even when the power of higher-frequency oscillations is modulated at the lower frequency of the sensory input, oscillations at the same frequency of the input can still be functionally important: they may coordinate rapidly changing and local neural processing, usually reflected by high-frequency brain activities, with brain activities operating at slower and more behaviorally relevant timescales [1,34,35].

Therefore, in the current study, we investigated whether the brain encodes long-timescale dynamics of painful input through entrainment of ultralow-frequency neural oscillations. Specifically, we investigated (1) whether and how amplitude and phase modulations contribute to such entrainment, (2) whether this entrainment is supramodal, and (3) whether the strength of entrainment reflects the variability of perceptual sensitivity across participants.

To address these questions, we recorded high-density EEG (128 electrodes) from participants receiving continuous thermo-nociceptive and continuous loud auditory stimuli oscillating at 0.1 Hz. Painful stimuli were delivered over the hand dorsum using a feedback-controlled laser stimulator with high temporal and temperature precision. Participants were requested to rate the perceived stimulus intensity on a visual analogue scale (VAS). To control for the confounding effect of the rating task, in two additional conditions participants received painful and auditory stimuli but were not required to rate them. Finally, to control for stimulus-intensity effects, we included an additional condition in which painful stimuli of lower intensity were delivered. Thus, there were five conditions in total.

Our results provide clear evidence that the long-timescale dynamics of nociceptive input are encoded by neural oscillations at the same ultralow frequency of the input. This ultralow-frequency entrainment was not supramodal, as it was robust during nociceptive stimulation but not present during auditory stimulation of similar intensity. Remarkably, the strength of neural entrainment to the nociceptive input was predictive of pain sensitivity across individuals.

## Results

### Stimulus input and perceptual ratings have similar temporal profiles

**Fig 1:**
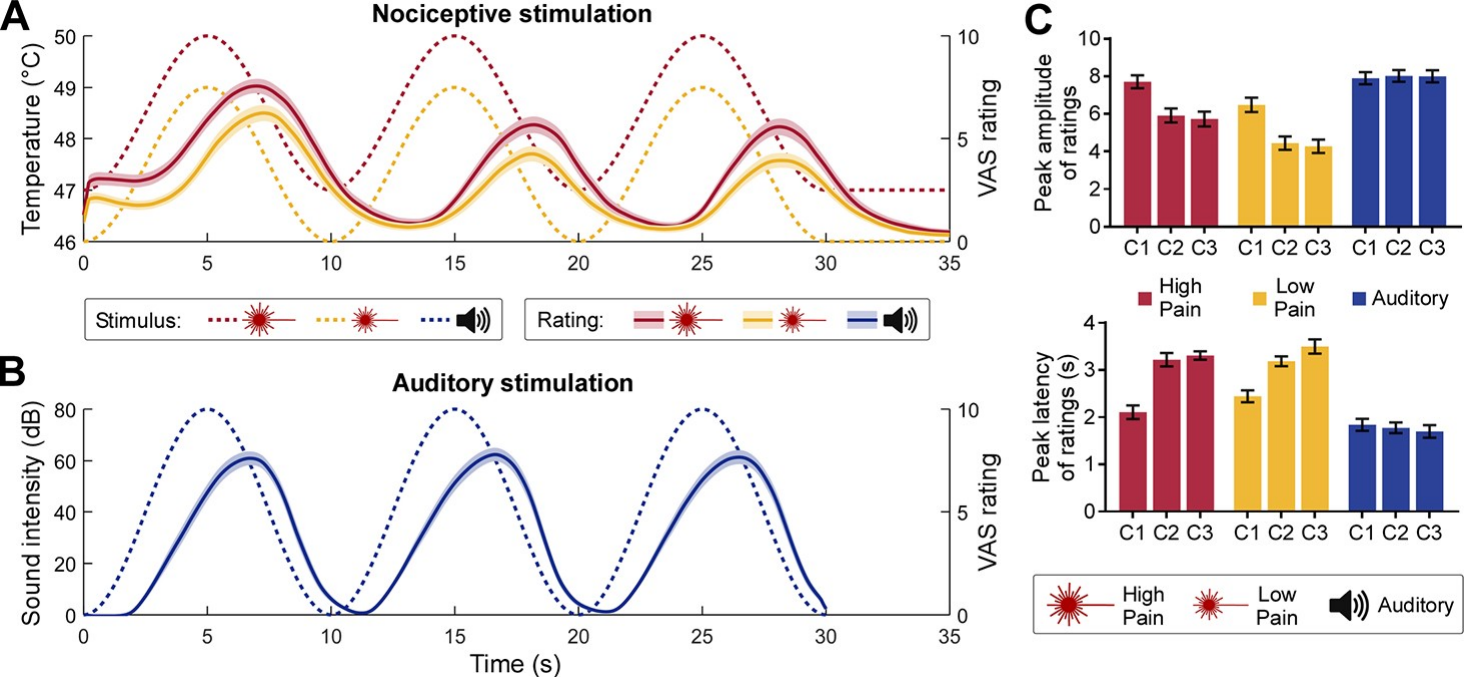
Rhythmic stimulus inputs and perceptual ratings. (**A**) Nociceptive stimuli consisted of a 0.1-Hz sinusoidal modulation of skin temperature with a 3°C difference between peaks and troughs, lasting 30 seconds. Stimulation temperatures were adjusted for each participant (see Materials and methods). High-pain stimuli (dotted red line) were set to 1°C above the low-pain stimuli (dotted yellow line). In some conditions, participants (*N* = 30) were required to continuously rate their perceived pain intensity using a VAS ranging from 0 to 10. Both the high-pain (solid red line) and low-pain (solid yellow line) rating time courses followed the nociceptive input (shaded regions indicate the SEM across participants). (**B**) Auditory stimuli consisted of a 0.1-Hz sinusoidal modulation of the amplitude of a 280-Hz pure tone (dotted blue line). In some conditions, participants were required to continuously rate their perceived sound intensity (solid blue line). (**C**) Peak amplitude (top) and latency (bottom) of the intensity ratings. The peak latency is expressed as difference between a peak in the rating and the corresponding peak in the stimulus. Error bars indicate the SEM across participants. Data underlying these plots can be found in S1 Data. C1-C3, cycle 1 to 3; SEM, standard error of the mean; VAS, visual analogue scale.

Participants’ continuous rating of perceived intensity roughly followed the temporal pattern of the rhythmic stimulation. In both pain (Fig 1A) and auditory (Fig 1B) rating time series, we observed three cycles whose period was similar to that of the stimulus. We formally compared the peak amplitude and latency of ratings across conditions and cycles. The results (Fig 1C and S1 Text) showed that auditory ratings peaked earlier than pain ratings in all three cycles and that pain ratings were decreased and delayed in the last two cycles compared to the first one (Fig 1C).

### Low-frequency nociceptive input enhances neural activity at the frequency of the stimulus

**Fig 2:**
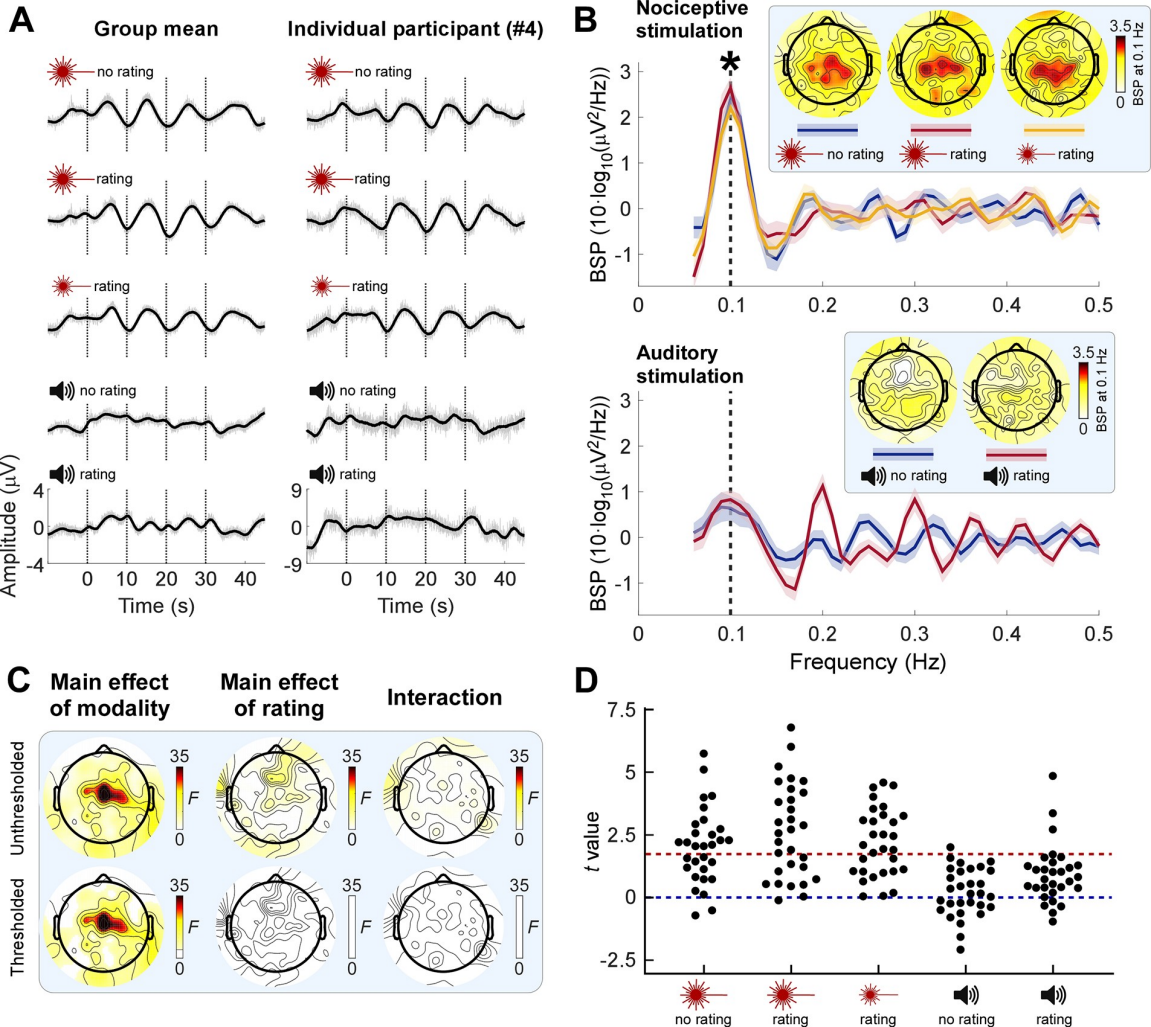
Neural oscillations are enhanced at the frequency of nociceptive input. (**A**) EEG time series averaged across participants (left column; *N* = 30) and from a representative participant (right column) in a central electrode cluster (Cz and its closest neighbors FCC1h, FCC2h, CCP1h, and CCP2h). Note how the neural activity displayed an oscillatory pattern reminiscent of that of sensory input in pain but not in auditory conditions. Signals smoothed with a moving mean filter with a 2-second window (black) are superimposed on unsmoothed signals (gray). (**B**) BSP of EEG in the central electrode cluster during nociceptive (top) and auditory (bottom) stimulation. Shaded regions around the solid lines indicate SEM across participants. Consistent power enhancement across participants (marked by asterisk; one-sample *t* test of BSP against 0, FDR corrected across frequencies) was only observed at the stimulus frequency (0.1 Hz, vertical dashed line) during nociceptive stimulation. Insets show the scalp topographies of the BSP at 0.1 Hz. (**C**) Topographies of F values from a two-way repeated-measures ANOVA exploring the effect of factors Modality and Rating on 0.1-Hz BSP. Top row: F values; bottom row: thresholded F values (*P* < 0.05, FDR corrected across electrodes); nonsignificant regions are masked with white. (**D**) Single-participant *t* values expressing the across-trial consistency of 0.1-Hz power enhancement at central electrodes. Red dashed line: *t* value at *P* = 0.05. Data underlying these plots can be found in S1 Data. BSP, background-subtracted power; EEG, electroencephalography; FDR, false discovery rate; SEM, standard error of the mean.

Next, we examined whether brain activities encoded the low-frequency rhythmic stimulation. When inspecting the time-domain EEG responses to the nociceptive input, we observed that central electrodes displayed a clear oscillatory pattern reminiscent of that of the stimulus (Fig 2A; see also S1 Fig for EEG time series at other electrodes). Importantly, these EEG oscillations were not visible in response to the auditory stimulation. To quantify the frequency contents of these EEG responses, we transformed single-trial EEG signals into the frequency domain and then averaged the resultant power spectra across trials, for each participant and condition (S2 Fig). Note that we performed the frequency decomposition at trial level rather than on single-participant average waveforms because in the former case, it is possible to detect an increase of power regardless of whether neural activity is phase-locked across trials. To reveal the frequency of power increases, we subtracted the average power of neighboring frequencies from each given frequency, yielding background-subtracted power (BSP), as previously recommended [20,36,37]. We found strong evidence for a power enhancement only at 0.1 Hz for all three conditions entailing nociceptive stimulation (Fig 2B; all *t*_29_ > 7.802, *P* < 0.0001, Cohen’s *d* > 1.424). This effect was maximal in central scalp regions (Fig 2B; also see S3 Fig for detailed results at other frequencies and scalp positions). This power enhancement could be consistently detected across single trials in the majority of individuals (Fig 2D).

We observed strong evidence that the BSP at 0.1 Hz in central scalp regions was greater in pain than in the auditory conditions (main effect of Modality: *F*_1,29_ = 39.48, *P* < 0.0001, partial *η*^2^ = 0.5765, two-way repeated-measures ANOVA at Cz; see Fig 2C for scalp topography of this effect). There was no evidence for a main effect of Rating or for a Modality × Rating interaction (Fig 2C), indicating that the power enhancement was not dependent on the rating task.

These results showed an enhancement of neural activities specifically at the frequency of rhythmic painful stimulation. Importantly, this effect was neither supramodal (i.e., it was not present in audition) nor dependent on whether the participants were performing a rating task. Also, the observation of an increase of BSP calculated using an extremely narrow frequency window of 0.06 Hz (i.e., from −0.03 Hz to +0.03 Hz with respect to 0.1 Hz) strongly indicates that the observed power increase is not consequent to a general autonomic response but has instead a neural origin.

### Phase reorganization of neural activity at the frequency of the nociceptive stimulus

**Fig 3:**
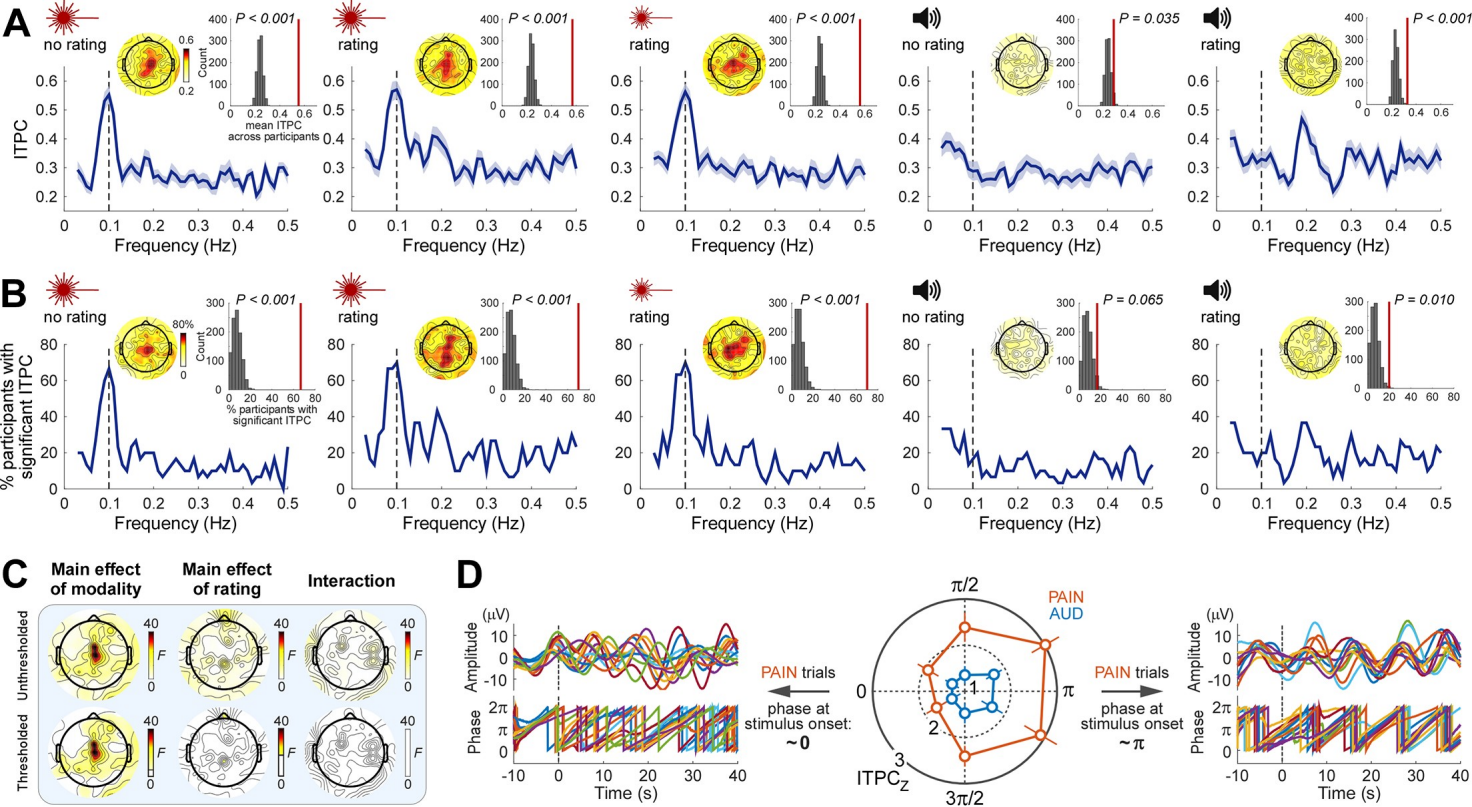
Rhythmic nociceptive input adjusts the phase of neural oscillations at stimulus frequency. (**A**) ITPC averaged across participants (*N* = 30) as a function of frequency at central electrodes (blue lines; shaded regions indicate SEM across participants). Note that pain conditions show an ITPC peak at the stimulus frequency (0.1 Hz, vertical dashed line), whereas the auditory conditions do not. Insets show topographies of the ITPC at 0.1 Hz and the comparison of the 0.1-Hz ITPC from central electrodes (vertical red lines) to ITPC obtained from randomized data. (**B**) Percentage of participants with significant ITPC (Rayleigh’s test *P* < 0.05) as a function of frequency at central electrodes. Note the peak at 0.1 Hz during nociceptive but not auditory stimulation. Insets show topographies of the percentage of participants with a significant ITPC at 0.1 Hz and the comparison at central electrodes to the same percentage obtained from randomized data. (**C**) Topographies of F values from a two-way repeated-measures ANOVA exploring the effect of factors Modality and Rating on 0.1-Hz ITPC. Top row: F values; bottom row: thresholded F values (*P* < 0.05, FDR corrected across electrodes); nonsignificant regions are masked with white. (**D**) The degree of phase locking during nociceptive stimulation was dependent on the phase of prestimulus oscillations. The strongest phase locking occurred in trials in which the stimulus started around the trough (π rad) of ongoing oscillations (right: pain trials in the two bins around π from a representative individual), whereas the weakest phase locking occurred when the stimulus started around the peak (0 rad) of ongoing oscillations (left: pain trials in the two bins around 0 from the same individual). As the signals displayed in the left and right panels are subgroups of trials selected for having a certain prestimulus phase, the prestimulus phases shown in each panel are unavoidably aligned. Error bars indicate SEM across participants. Data underlying these plots can be found in S1 Data. FDR, false discovery rate; ITPC, intertrial phase coherence; SEM, standard error of the mean.

The finding that neural activity and stimulus profile have the same peak frequency does not necessarily indicate a stable phase relationship between the two [38]. To test for such a phase relationship, we quantified the EEG phase locking across trials using intertrial phase coherence (ITPC), separately for each participant and condition. Since EEG trials were time aligned to the onset of the rhythmic stimulation, it follows that here ITPC also quantified the consistency of the phase relationship between stimulus and EEG data within each individual. We observed that during painful, but not auditory, stimulation there was a clear peak of ITPC at 0.1 Hz. This was observed in both the mean ITPC across participants (Fig 3A) and the percentage of participants with significant ITPC (Fig 3B; ITPC significance was determined in each participant using the Rayleigh’s test for circular uniformity [39]). This phase-locking effect at the frequency of the nociceptive stimulation was maximal in central scalp regions (Fig 3A and 3B). To test for the group-level consistency of this effect, we compared the group-mean ITPC and the percentage of participants with significant ITPC to randomized data (see Materials and methods). In all pain conditions, both ITPC measures at 0.1 Hz in central regions were consistently greater than chance (all *P* < 0.001) (Fig 3A and 3B; also see S4 Fig for detailed test results at other frequencies and positions).

We observed strong evidence that the 0.1-Hz ITPC in central scalp regions was higher in pain than in audition (main effect of Modality: *F*_1,29_ = 40.15, *P* < 0.0001, partial *η*^2^ = 0.5806, two-way repeated-measures ANOVA at Cz; see Fig 3C for scalp topography of this effect). There was no main effect of Rating on 0.1-Hz ITPC, except in two electrodes distant from each other (Fpz, CPz), in which the evidence for this main effect was, however, weak (Fig 3C for scalp topography of this effect). There was no Modality × Rating interaction (Fig 3C).

Given that phase locking across trials (i.e., a relatively stable phase relationship between the neural responses and the stimulus profile) could result from a reorganization of the phase of ongoing neural oscillations, we tested whether the degree of phase locking was related to the phase of ongoing EEG. We sorted single trials into six bins according to the prestimulus phase at central scalp regions and calculated 0.1-Hz phase locking (ITPC_Z_; Rayleigh’s Z, see Materials and methods) during stimulation for each bin, modality, and participant. As shown in Fig 3D, we observed clear evidence that the phase of prestimulus oscillations influenced the degree of phase locking during stimulation. Phase locking in the pain trials was maximal when the onset of the rhythmic nociceptive stimulation coincided with the trough of ongoing oscillations (i.e., around π) and minimal when the stimulus onset coincided with the peak (i.e., around 0 or 2π) (*F*_5,145_ = 4.433, *P* = 0.0009, partial *η*^2^ = 0.1326, two-way repeated-measures ANOVA, main effect of Bin; post hoc tests showed significant differences between either of the two bins around π and either of the two bins around 0, all *P* < 0.0054). Although there was strong evidence that the phase locking in auditory trials was lower than that in pain trials (*F*_1,29_ = 18.31, *P* = 0.0002, partial *η*^2^ = 0.3870, main effect of Modality), the lack of a Bin × Modality interaction (*F*_5,145_ = 1.018, *P* = 0.4094, partial *η*^2^ = 0.0339) indicated that also in the auditory trials, phase locking was influenced by the phase of prestimulus oscillations, although to a lesser extent (there was no post hoc evidence for differences in phase locking between bins in auditory trials; *P* > 0.1440 in all bin comparisons).

To further illustrate the building up of phase locking over time, we also calculated ITPC using the instantaneous phase of ultralow-frequency oscillations (see Materials and methods). In all three pain conditions, instantaneous ITPC increased after stimulus onset, whereas in the two auditory conditions, ITPC remained at prestimulus level (S5 Fig).

Altogether, these results show phase locking of neural oscillations at the frequency of the rhythmic nociceptive stimulation. The degree of such phase locking clearly depended on the phase of ongoing neural oscillations. Similar to the power increase, this phase-locking effect was also not supramodal and not related to whether the participants were performing a rating task.

### Pain sensitivity across individuals is reflected in the strength of neural entrainment

**Fig 4:**
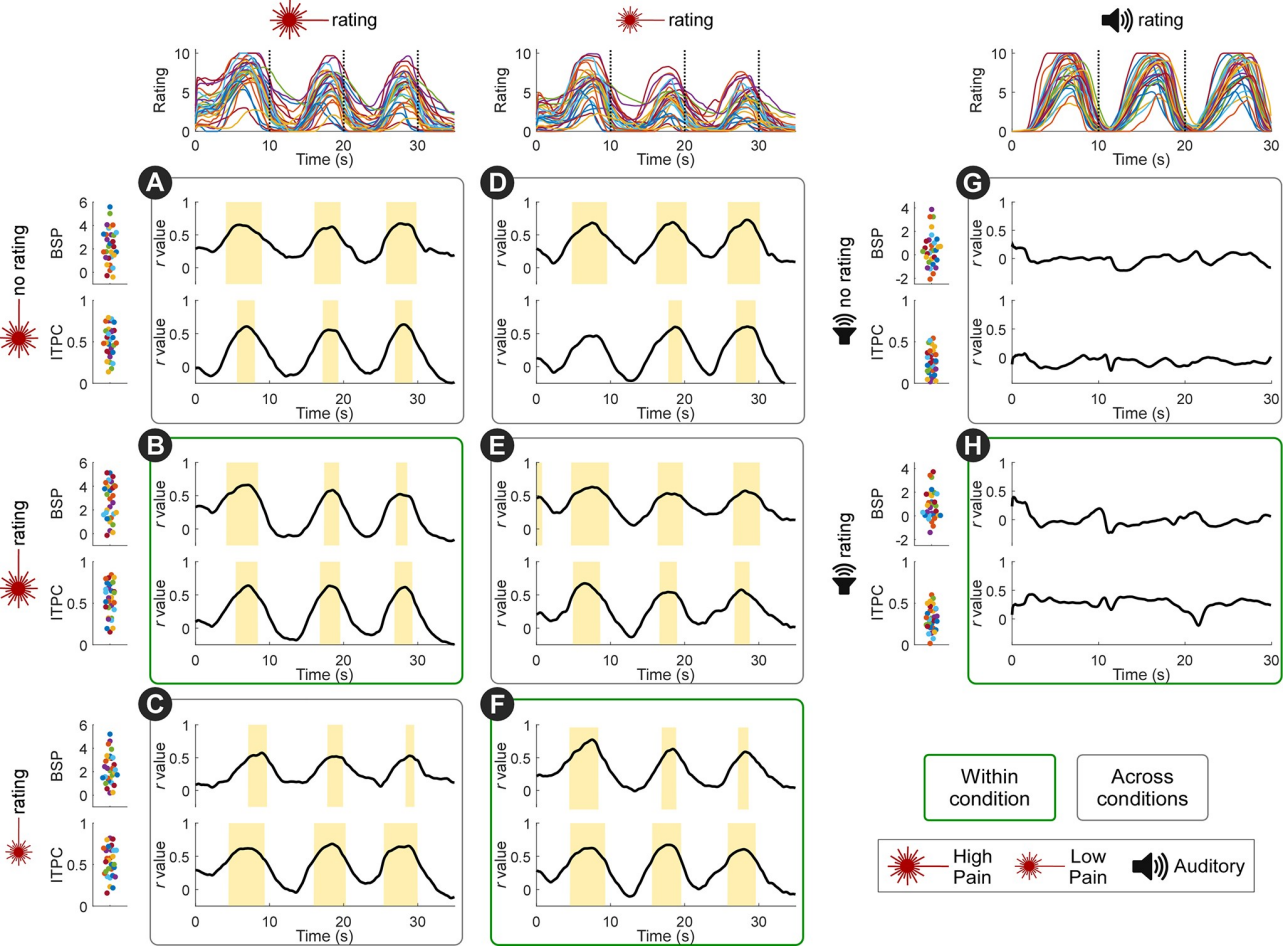
Pain sensitivity across individuals is reflected in the strength of neural entrainment: Power and phase locking. Traces on the top show individual rating time series. Scatterplots on the left show individual BSP (top scatterplots of each condition, expressed as 10∙log_10_ [μV^2^/Hz]) and ITPC (bottom scatterplots of each condition) at 0.1 Hz at central electrodes. Black lines show the correlation coefficients between BSP/ITPC and ratings across time. Both BSP and ITPC of each of the three pain conditions were correlated to the high-pain (**A-C**) and low-pain (**D-F**) ratings, in the time intervals around the rating peaks (marked by yellow bars; *P* < 0.05, FDR corrected across time points). Note that the positive across-participant correlation was present not only when correlating BSP and ITPC with ratings within condition (**B,F**), but also when correlating BSP and ITPC with ratings across conditions (e.g., when correlating BSP and ITPC from the pain no-rating condition with high-pain and low-pain ratings; **A,D**). Importantly, BSP and ITPC in the auditory conditions were not correlated to the auditory intensity ratings (**G-H**). *N* = 30 participants. Data underlying these plots can be found in S1 Data. BSP, background-subtracted power; FDR, false discovery rate; ITPC, intertrial phase coherence.

**Fig 5:**
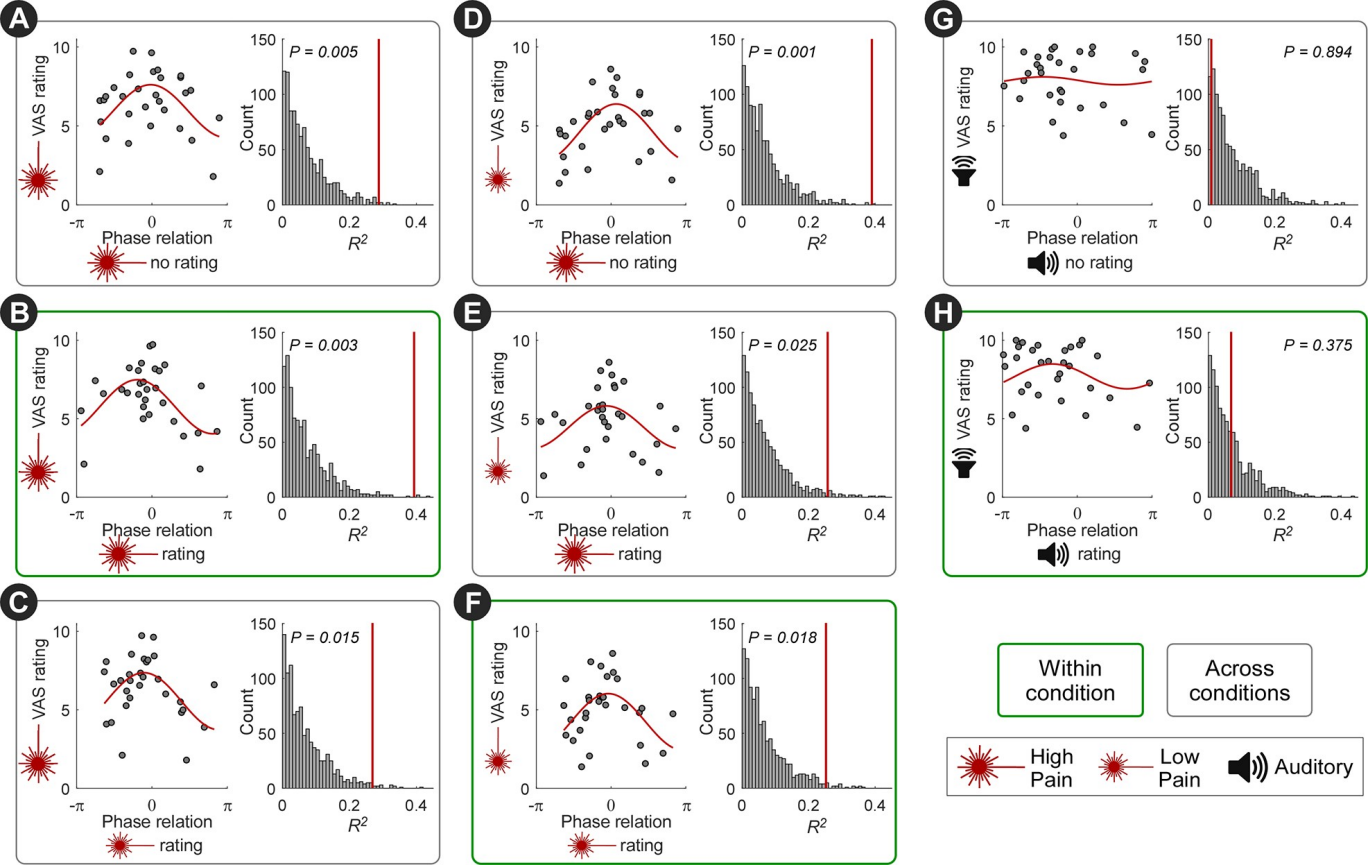
Pain sensitivity across individuals is reflected in the strength of neural entrainment: Phase. The across-participants variability in perceived pain intensity was also reflected in the phase relationship between the entrained oscillations and the stimulus profile. For each individual, the mean of high-pain (**A-C**), low-pain (**D-F**), or auditory (**G-H**) peak ratings was plotted against the phase difference between the 0.1-Hz oscillation at central electrodes and the stimulus (“phase relation”). To evaluate this relationship, we fitted a single-cycle cosine function (red lines). Coefficient of determination (*R*^2^) of the cosine fit was tested by random permutation of the phase across participants. Individuals who rated the nociceptive stimulus as more painful entrained more closely to the phase of the nociceptive input (i.e., with a phase relation around 0). This relationship was preserved not only within condition (**B,F**) but also across conditions (**A,C-E**). Importantly, such relationship was not present in the auditory conditions (**G-H**). *N* = 30 participants. Data underlying these plots can be found in S1 Data. VAS, visual analogue scale.

Perceived pain was stronger in participants with a stronger neural entrainment. Specifically, we correlated the pain rating time series to the indices of power enhancement (BSP) and phase locking (ITPC) at 0.1 Hz in central scalp regions. We observed clear positive correlations in the time interval around each rating peak (Fig 4). Remarkably, the across-participant variability in perceived pain intensity was also reflected in the phase relationship between the entrained oscillations and the stimulus (Fig 5). To evaluate this relationship, we fitted a single-cycle cosine to the participant-mean peak rating as a function of the phase of 0.1-Hz oscillation in central scalp regions. In individuals who rated the nociceptive stimulus as more painful, the phase of neural activity at 0.1 Hz was closer to that of the stimulus input. This is an important finding, given that almost all nociceptive-evoked neural responses fail to track pain sensitivity across participants, in both human and animal studies [40].

Importantly, all these relationships (i.e., the correlation between BSP/ITPC and pain ratings, and the relationship between phase and pain ratings) existed not only (1) within the conditions entailing nociceptive stimulation with ratings (Fig 4B and 4F; Fig 5B and 5F) but also (2) between high-pain and low-pain conditions (Fig 4C, 4D and 4E; Fig 5C, 5D and 5E) and even (3) between pain ratings and the neural entrainment in the pain condition without ratings (Fig 4A and 4D; Fig 5A and 5D). The relationships in (2) suggested that the strength of neural entrainment reflected individual pain sensitivity. The relationships in (3) further demonstrated that the link between pain sensitivity and the strength of neural entrainment was not driven by the rating task. No such relationships were observed in the conditions entailing auditory stimulation (Fig 4G and 4H; Fig 5G and 5H).

To test whether the variability in stimulus temperature contributed to the above results, we also tested for an across-participants relationship between stimulus temperature and pain ratings, as well as between stimulus temperature and neural entrainment. We did not observe such relationships (see S1 Table for detailed statistical results).

Having found clear correlations between pain ratings and three features of the entrained oscillations (power enhancement, phase locking, and phase difference between brain oscillations and stimulus), we further investigated the contribution of each of these variables to explain the pain rating variance across participants. Specifically, we performed multiple linear regression and used the Akaike information criterion (AIC) [41] and the Akaike weights (w_i_) [42] to compare regression models containing different combinations of the three explanatory variables (see Materials and methods). These analyses were performed for each pain condition and separately for the ratings of the high-pain and low-pain stimulation as the dependent variable. The results, summarized in S3 Table, showed no obvious consistency in which model explained most of the rating variance, although the best models almost always entailed a combination of measures of power and phase.

Taken together, these results show that the strength of entrainment could predict the sensitivity of each participant to painful stimulations.

### Stimulus-induced neural oscillations outlast nociceptive input

**Fig 6:**
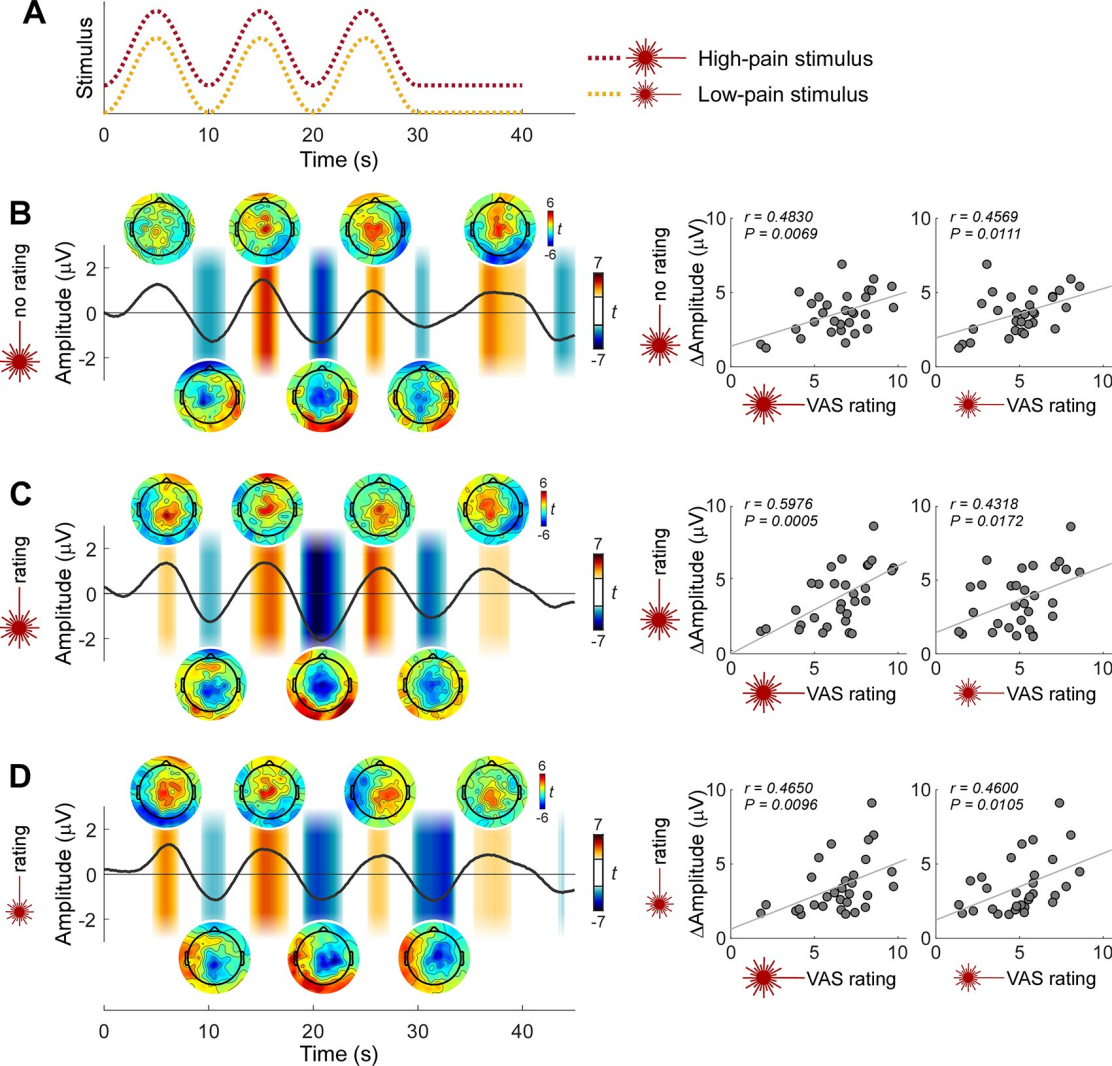
Stimulus-induced neural oscillations outlast the rhythmic nociceptive input. The observation that neural oscillations around 0.1 Hz continued after the end of the rhythmic nociceptive input provides additional evidence for a real neural entrainment of ongoing EEG activity. (**A**) Temporal profile of the high-pain (red dotted line) and low-pain (yellow dotted line) stimulation. (**B-D**) EEG signal at central electrodes was smoothed with a moving mean filter with a 2-second window, linearly detrended, and finally averaged across participants (*N* = 30) in the conditions entailing high-pain stimulation without rating task (**B**), high-pain stimulation with rating task (**C**), and low-pain stimulation with rating task (**D**). Shaded regions indicate the time windows in which the EEG amplitude is significantly different from 0 (*P* < 0.05, point-by-point one-sample *t* test against 0, FDR corrected across time points). Scalp maps show the *t* value topographies within 1-second window around the peak and trough of each cycle. The similarity of scalp topographies at the peak of the cycle after the end of rhythmic stimulation with those of the previous cycles further indicates self-sustained activity of the same underlying neural process. Plots on the right show that the amplitude difference between the peak and trough of the cycle after the end of rhythmic stimulation was correlated to the mean of the peak pain ratings across participants. Data underlying these plots can be found in S1 Data. EEG, electroencephalography; FDR, false discovery rate; VAS, visual analogue scale.

A remarkable observation was that neural oscillations around 0.1 Hz continued for at least 10 seconds after the end of the rhythmic nociceptive input (Figs 2A and 6). The observation that the scalp topographies at the peak of the additional cycle resembled those of the previous cycles (Fig 6) further indicates self-sustained activity of the same underlying neural process. These observations also provide further evidence that the observed ultralow-frequency oscillations were not consequent to autonomic responses. Finally, the amplitude of this additional oscillation after the end of rhythmic nociceptive stimulation was correlated with pain ratings across participants (Fig 6).

### Source modeling of ultralow-frequency oscillations

We estimated the brain generators of the amplitude of ultralow-frequency oscillations using CLARA (classical LORETA analysis recursively applied), a distributed source analysis approach [43–45]. In all three pain conditions, CLARA estimated two distinct sources, with the strongest source consistently located in the posterior cingulate cortex (PCC) (high-pain no rating: 4, −45, 31 mm [Talairach coordinates at the maximum intensity], maximum intensity 2.1 nAm/cm^3^; high-pain rating: −10, −39, 35 mm, 2.4 nAm/cm^3^; low-pain rating: −4, −45, 24 mm, 1.7 nAm/cm^3^) (S6 Fig). In all three conditions, a second, weaker source was located at the boundary between the insula and putamen (high-pain no rating: 25, 4, −4 mm, 1.3 nAm/cm^3^; high-pain rating: −25, 11, 3 mm, 1.2 nAm/cm^3^; low-pain rating: −25, 11, 10 mm, 1.3 nAm/cm^3^). The side of this second source, however, was different in the three conditions: in the no-rating condition, it was located on the right hemisphere (i.e., contralateral to the hand receiving the nociceptive stimulation), whereas in the rating conditions, it was located on the left hemisphere (i.e., contralateral to the hand rating the perceived intensity).

## Discussion

Here, we aimed to identify the neural activity present during tonic sensory stimuli that produce slowly fluctuating sensations. We delivered intensity-matched auditory and painful stimuli fluctuating at 0.1 Hz and observed that only painful stimuli resulted in both a power enhancement and a phase locking of brain activity at the same frequency of the stimulus (Figs 2 and 3). Thus, this brain response was not supramodal and was possibly selective for the somatosensory system. This response could reflect a true neural entrainment, since (1) the degree of phase locking depended on the phase of ongoing brain oscillations occurring before the onset of the rhythmic input (Fig 3D), and (2) the stimulus-induced brain oscillations outlasted the rhythmic input (Fig 6). Importantly, this neural entrainment to the rhythmic painful input was not due to the rating task, as it was also present when participants did not have to rate the painfulness of the stimuli (Figs 2 and 3). Finally, the strength of the neural entrainment was correlated with pain sensitivity across individuals (Figs 4 and 5), a relationship that persisted even in the neural activity outlasting the rhythmic stimulus (Fig 6).

These findings show that the brain encodes long-timescale dynamics of nociceptive input through entrainment of ultralow-frequency neural oscillations. This work not only represents a step toward analyzing brain processes in more clinically relevant models of long-lasting and dynamic pain [15,16] but also sheds new light on the functional significance of neural oscillations at frequencies well below the traditional boundaries of human EEG rhythms.

### Neural entrainment or evoked responses?

The power enhancement (Fig 2) and phase locking (Fig 3) of neural activities observed at the frequency of nociceptive input could be explained by either repeated evoked responses or an entrainment of ongoing neural oscillations to the rhythmic stimulus [6,7,46–52]. Albeit important, distinguishing between these two different mechanisms is notoriously difficult, and claims of entrainment are, unsurprisingly, highly debated [6,49].

Although specifically designed studies will be needed to adequately address the question of whether the oscillations we observed reflect neural entrainment, our findings point toward this interpretation. First, we found clear evidence that the degree of phase locking depended on the phase of ongoing neural oscillations occurring before stimulus onset (Fig 3D). Indeed, such phase interactions are predicted by theoretical models and experimental data of entrainment of neural oscillators: although rhythmic stimuli drive brain activities, the effectiveness of this process, reflected in the phase reorganization, also depends on the state of the neural oscillators [33,38,53,54]. Second, as depicted in Fig 6, the neural oscillations outlasted the rhythmic painful input. Furthermore, the similarity of scalp topographies of the oscillations during and after rhythmic stimulation suggests common neural generators. This interpretation is corroborated by both theoretical and experimental studies [7,8,27,32,33,55–57] demonstrating that entrained neural oscillations can be self-sustaining for a certain amount of time after the end of external rhythm. Future experiments collecting poststimulus data for longer time intervals will allow a more accurate characterization of the self-sustained activity. Such experiments would also illustrate the recovery process of the oscillatory system from the entrained state.

This possible neural entrainment could reflect processing of nociceptive information that is not captured by more widely used paradigms employing transient stimuli. Indeed, unlike transient responses that are discrete in time, the entrained oscillations are continuous and might reflect an adaptive internal model of the input temporal regularities, which could facilitate sensory processing of incoming events [1,2].

### Neural entrainment to rhythmic painful input is independent of rating task

We observed neural entrainment to the rhythmic painful input regardless of whether participants were required to continuously rate pain intensity (Figs 2, 3 and 6, S3 Fig and S4 Fig).

Continuous perceptual ratings are commonly used to investigate the neural correlates of percepts occurring at long timescales (e.g., tonic experimental pain in healthy volunteers [20,22,25] or spontaneous fluctuations of pain in chronic pain patients [12,13]). Such continuous ratings are typically obtained with a finger-span device [12,13,22,25] or a slider [20]. Although such ratings provide valuable information on the dynamic fluctuations of perception, they heavily confound the analysis of neural data because of the superposition of brain activities related to the motor and cognitive activity related to the rating task [58]. A strategy to control for this confound is to ask participants to continuously rate the perceived intensity of stimuli belonging to another sensory modality (e.g., vision) [12,13,22,25,59]: the brain activity measured during painful but not visual stimulation should then reflect pain-selective activity. This paradigm assesses whether the rating task is sufficient to explain the brain response sampled during painful stimulation. However, it cannot resolve whether the same brain response remains when no rating task is performed—that is, whether the rating task is necessary for observing the brain response. To effectively address this issue, we included in our experiments additional control conditions in which participants had to rate the intensity of neither the auditory nor the painful stimuli.

Thus, our results demonstrate that rating-related brain activities were not necessary for the observed entrainment of brain oscillations to the rhythmic painful input. Indeed, the power enhancement, the phase locking, and the continuation of the entrained neural oscillations after stimulus offset were also present in the pain condition not involving the rating task (Figs 2, 3 and 6, S3 Fig and S4 Fig).

Although we did not observe strong evidence for an effect of rating task at the stimulation frequency of 0.1 Hz for either power or phase, the task of rating auditory stimuli enhanced the power and the phase locking at 0.2 and 0.3 Hz (Figs 2B, 3A and 3B and S4 Fig). In contrast, rating of painful stimuli only slightly enhanced the phase locking at 0.2 and 0.3 Hz (S4 Fig). This frequency and modality pattern suggests that the rating effect reflects a different mechanism than the neural entrainment to the rhythmic input [48]. The fact that the increases and decreases were more regular in auditory than in pain ratings (Fig 1) might explain why the effect of rating auditory stimuli was more evident.

It is worth noting that, although the continuous intensity ratings tracked the temporal profile of the rhythmic stimulation, they were generally delayed compared to the stimulus (Fig 1). This was at least in part a natural consequence of our experimental task, in which participants were asked to rate the intensity of the sensation elicited by the stimulus: the rating should, by definition, occur after the stimulus has been perceived. This makes our experiment different from the paradigms in which participants are asked to tap to, for example, musical beats [60], resulting in movements aligned to or even preceding each beat.

### Is the observed neural entrainment modality-specific?

As we discussed above, the entrainment of ultralow-frequency brain oscillations to stimulus input was clearly not supramodal, given that (1) the power increase of EEG signal at stimulus frequency was only consistently observed during nociceptive stimulation but not during loud auditory stimulation (Fig 2), and (2) only the EEG signals during nociceptive stimulation showed a predominant peak of phase locking at stimulus frequency (Fig 3). Importantly, these findings by no means imply that the entrainment we observed during nociceptive stimulation was modality-specific. Indeed, strictly speaking, demonstrating the pain specificity of a neural response is virtually impossible, as it would require testing all stimuli that could, in principle, elicit that response and show that that response only occurs when pain is experienced [16]. Instead, the current findings provide evidence that entrainment at the ultralow frequency used in this study occurs preferentially in response to somatosensory input. That the observed entrainment is preferential to pain would require testing whether it occurs less strongly during non-nociceptive somatosensory stimulation.

The lack of entrainment to the auditory stimuli is not trivial, since there is a considerable amount of evidence for entrainment of neural oscillations to rhythmic auditory stimulation [4,7,30,31,33,50]. This lack of entrainment to auditory input is unlikely to be a false negative, as we have applied the same analysis pipeline to all experimental conditions from both modalities and explored the results across all scalp electrodes. A possible explanation is that the frequency of the delivered auditory stimulation is substantially lower than the timescale of dynamics for which the auditory neural circuits are optimized. Indeed, the temporal structures of speech and music largely occur in the subsecond range [3,61]. Accordingly, previous evidence for auditory entrainment is primarily observed during stimulation at delta (0.5–4 Hz) and theta (4–8 Hz) frequencies [7,8,30,33,62,63]. It is worth mentioning that there are previous reports of auditory entrainment to, for example, tone pips [64], speech [65], or higher-order rhythms [66,67] occurring at frequencies between 0.5 and 1 Hz. Still, to the best of our knowledge, there is no evidence of auditory entrainment below 0.5 Hz.

These previous observations, together with the current results, suggest a different frequency preference for oscillatory entrainment across sensory systems [68]. Why would the nociceptive system respond to lower ranges of stimulus frequencies than the auditory system? Given that the temporal dynamics of fluctuations of spontaneous pain and somatosensory detection occur at very low frequencies [11,69], the brain representations of long-timescale variability might be more essential and relevant to the processing of pain information. These considerations suggest that neural entrainment is tuned to the temporal scale of the statistical regularities characteristic of different sensory modalities, a hypothesis that would require further testing.

As a final remark, we note that some previous studies have shown that auditory entrainment is task-dependent and requires top-down attention [7,31,70–72]. This makes the lack of entrainment in our auditory rating condition more interesting, as high-intensity auditory stimuli were presented, and the rating task required continuous attention. Still, we cannot rule out that in the presence of a more demanding task, auditory entrainment may appear, or that, regardless of task, the stimulus parameters we used did not allow for auditory entrainment. Thus, an interesting challenge for future research will be to test whether and how the effects of task or attention change with stimulus parameters. It is also interesting that pain drives entrainment regardless of task, whereas audition might need a task of a certain difficulty to do so—an admittedly post hoc possible explanation is that pain is more necessarily related to action [73].

### The strength of neural entrainment reflects pain sensitivity across individuals

We observed a clear relationship between perceived pain intensity and the strength of neural entrainment to the nociceptive input. It is important to highlight that this is an across-participants relationship. Therefore, the presence of an oscillation at 0.1 Hz at a specific time point does not necessarily indicate that there is, at that same time, pain. This is exemplified by the fourth cycle of the entrained oscillations (Fig 6), occurring at a time when pain was close to zero. Rather, we found that individuals with higher pain sensitivity had greater power enhancement and phase locking at the frequency of the nociceptive input (Fig 4), as well as a phase of the entrained oscillations closer to the phase of the nociceptive input (Fig 5). This is a remarkable result, for two reasons. First, even the commonly observed within-participant correlation between nociceptive-evoked responses and subjective pain ratings have been shown to be not obligatory—i.e., they can be easily disrupted by a large number of experimental manipulations (e.g., expectation, stimulus repetition, presentation of nonpainful but isosalient stimuli, congenital insensitivity to pain; [74–76]). Second, almost all nociceptive-evoked neural responses fail to track pain sensitivity across participants, in both human and animal studies [40].

It is important to note that the relationship between entrainment and pain sensitivity across participants was not driven by the rating task and was also present when examining conditions entailing different intensities of nociceptive stimulation. Most notably, the relationship between oscillatory amplitude and ratings persisted following the end of rhythmic stimulation (Fig 6). Thus, the strength of neural entrainment to rhythmic nociceptive input is, in these experimental conditions, a neural marker of pain sensitivity across individuals.

For clinical purposes, it is important to predict pain across different individuals [77–80]. For example, pain may need to be inferred for new patients when verbal reports are unavailable or unreliable, to optimize analgesic treatment and general care [79]. Including ultralow-frequency neural entrainment in future pain prediction models may allow for better pain prediction between individuals, especially for the more realistic situations in which pain fluctuates as an ongoing percept.

Relevant to this discourse, a recent study revealed that brain oscillations in the gamma band index the variability of pain sensitivity across individuals, in both humans and rodents [40]. However, the clinical relevance of that observation is limited because (1) gamma oscillations were elicited by transient nociceptive stimuli causing painful sensations barely reflecting clinical pain, and (2) because of low signal-to-noise ratio, gamma oscillations are hardly detected in EEG recordings from single individuals. The ultralow-frequency oscillations we describe here, in contrast, (1) are elicited by continuous and fluctuating nociceptive input causing ongoing pain that better mimics spontaneous pain in patients and (2) can be detected in most single participants (Figs 2D and 3B). Despite these distinctions, these high- and low-frequency indicators of pain sensitivity across individuals could be related. Therefore, an interesting direction for future research is to investigate whether the phase/frequency modulation of slow oscillations and the power modulation of gamma (or other fast) oscillations work synergistically, e.g., to form a hierarchical structure supporting different spatial/temporal scales of brain operation. This could be relevant to tracking and predicting the dynamics of pain, especially in clinical situations [11–13].

### Putative generators of the ultralow-frequency oscillations

Distributed source analysis revealed two brain generators of the stimulus-induced ultralow-frequency oscillations: a strong source in the PCC and a second, weaker source at the boundary between insula and putamen (S6 Fig). The possible functional significance of these sources is briefly discussed below, bearing in mind the inherent uncertainty of source modeling of scalp potentials. Indeed, given the infinite number of source configurations that can explain a certain distribution of scalp data, the conclusiveness of these results is limited unless corroborated by other imaging methods.

The most notable aspect of the source results is that the PCC, the main generator of the oscillations we described, is not part of the commonly-described generators of the transient responses elicited by short and fast-rising nociceptive stimuli: the anterior portion of the cingulate cortex, the bilateral operculoinsular cortex, and the contralateral primary somatosensory cortex [75,81]. This difference in source configuration is not surprising, if one considers that transient and fast-rising nociceptive stimuli evoke brain response reflecting the supramodal detection of salient events [75], a factor that we intended to avoid with the stimulation paradigm used in the current study.

PCC, traditionally considered a hub of the default mode network [82,83], has been linked to various cognitive functions (e.g., attention, learning, memory, reward processing; for reviews, see [84,85]). Pearson and colleagues [85] proposed a framework in which the PCC maintains and integrates information related to the statistical structure of dynamic environments and guides subsequent behavioral policy. This theory is in line with our interpretation that stimulus-induced ultralow-frequency oscillations reflect an adaptive internal model that retains the temporal regularities of sensory input and thereby allows the individual to meet the demands of a changing environment. The second, weaker source at the boundary between the insula and the putamen is consistent with the fact that the insula is one of the most frequently activated regions in pain imaging studies [86]. The effect of the rating task on the side of this second source suggests that its activity may also reflect motor planning or performance (which was necessary for our rating task), as speculated in [87].

## Materials and methods

### Ethics statement

The study was approved by the ethics committee of University College London (project ID 2492/001) and conducted according to the principles expressed in the Declaration of Helsinki. All participants gave written informed consent.

### Participants

Thirty healthy human volunteers (18 women; mean age ± SD, 22.8 ± 2.9 years, age range 19–30 years; all right-handed) participated in the study. They received monetary compensation for their participation. Before taking part in the experiment, participants were familiarized with the experimental setup and procedures.

### Experimental procedures

Throughout the experiment, participants were seated comfortably in front of a table in a silent, temperature-controlled room, with the palm of their left (nondominant) hand resting on the table. Each participant received tonic nociceptive stimuli on the dorsum of their left hand, as well as tonic auditory stimuli (see the Sensory stimuli section). In a number of trials of each stimulus type (15 out of 30 high-pain stimuli, 15 out of 15 low-pain stimuli, and 15 out of 30 auditory stimuli), participants were instructed to continuously rate their perceived stimulus intensity on a VAS ranging from 0 to 10 using a custom-built vertical slider controlled with their right hand. The slider was connected to a potentiometer to record their ratings. For nociceptive stimuli, the lower and upper ends of the slider were defined as “no pain” and “the maximum pain tolerable,” respectively. For the auditory stimuli, they were defined as “no sound” and “the loudest sound tolerable.” Ratings were recorded from the onset of the periodic stimulation and lasted for 30 seconds in auditory trials and 45 seconds in pain trials. Rating data were digitized at 1,024 Hz (USB-1408FS, Measurement Computing Corporation, Norton, MA, United States of America) and synchronized with stimulation triggers and EEG recordings. In all trials, participants were instructed to focus their attention on the stimuli and keep their gaze on a fixation cross placed centrally in front of them, at a distance of approximately 60 cm and 30° below eye level. The order of stimulus presentation and rating task is detailed in S4 Table. The experimenters started trials manually after ensuring that the participant was ready and instructed whether to rate the sensation. As a result, the time between the end of a trial and the beginning of the following trial ranged between 10.0 and 78.2 seconds (average 21.2 seconds). Participants were allowed to rest for approximately 2 minutes after every 15 trials.

### Sensory stimuli

Nociceptive tonic stimuli were generated by a temperature-controlled CO_2_ laser stimulator (Laser Stimulation Device, SIFEC, Ferrières, Belgium) with a wavelength of 10.6 μm and a beam diameter of 6 mm. The output power of the laser was continuously regulated by a feedback control loop based on an online monitoring of skin temperature at the target site [88]. The laser stimulation target was changed after each stimulus to avoid nociceptor fatigue, sensitization, and skin damage. Laser power modulation resulted in a 0.1-Hz sinusoidal modulation of skin temperature, starting with an initial phase of π (i.e., trough) and lasting for 30 seconds (Fig 1A). The temperature difference between peaks and troughs was 3°C. Each participant received laser stimuli of two intensity levels (high-pain and low-pain stimuli), individually adjusted as described below. To avoid saliency-related brain responses during the periodic stimulation, the skin temperature was first brought to the desired trough level in a 1-second heating ramp and then maintained at this level for 5 seconds before the onset of the periodic stimulation. After the periodic stimulation, the skin temperature was maintained at the trough level for 10 additional seconds.

Before the experiment, stimulus temperatures were determined individually as follows. First, pain detection threshold and pain tolerance were estimated. To measure the pain detection threshold, participants received linearly increasing stimuli at 1°C/second (with a cutoff at 54°C) on the dorsum of their left hand and were instructed to press a button with their right hand as soon as they felt a painful sensation. The button-press immediately terminated the stimulation. To measure the pain tolerance, participants were instructed to press the button as soon as they felt the painful sensation become intolerable. The laser target on the skin was changed after each stimulus. Both the pain detection threshold and pain tolerance were measured three times, in consecutive trials, and their corresponding temperatures were estimated as the mean of the three consecutive measurements. The trough temperature of the low-pain stimuli was set to 1°C above the pain detection threshold, and the trough temperature of the high-pain stimuli was set to 1°C above that of the low-pain stimuli. The peak temperature of the stimulus was always below the pain tolerance. The resulting peak temperature in the high-pain stimuli was 48.3°C ± 1.9°C (mean ± SD across participants).

Auditory stimuli were generated by MATLAB (MathWorks, Natick, MA, USA) and presented through headphones binaurally. Auditory stimuli consisted of a pure tone (frequency of 280 Hz) whose amplitude was sinusoidally modulated at 0.1 Hz for 30 seconds (Fig 1B). As for the nociceptive stimulation, the sinusoidal modulation started with an initial phase of π (i.e., trough). The sound intensity was individually adjusted to ensure that perceived intensity was similar to the high-pain condition. Auditory stimuli were presented using the Psychophysics Toolbox [89].

### Analysis of subjective sensations

Single-trial rating time series were down-sampled to 512 Hz and smoothed using a moving mean filter with a 1-second window. Filtered data were averaged across trials, for each participant and condition. The peak latency and amplitude of each rating cycle were measured from the average waveforms. The rating peak latencies were measured with respect to the corresponding peak latencies in the stimulus time series (i.e., 5, 15, and 25 seconds; Fig 1). A two-way repeated-measures ANOVA with factors Condition (three levels: high pain, low pain, and sound) and Cycle (three levels: cycle 1–3) was performed separately for the peak latency and amplitude. Post hoc paired-sample two-tailed *t* tests were performed when a significant (*P* < 0.05) main effect or interaction was found (false discovery rate [FDR] corrected; the same for post hoc tests described throughout the text).

### EEG recording and preprocessing

EEG was recorded continuously using 128 Ag/AgCl electrodes (SD-128, Micromed S.p.A., Treviso, Italy) placed on the scalp according to the 10–5 system. The EEG signal was sampled at 512 Hz, referenced to the nose, and filtered by an effective first-order, IIR causal high-pass filter at 0.02 Hz, which resulted from a combination of physical and software filters, as implemented in the Micromed system. Electrooculographic signals were simultaneously recorded using two surface Ag/AgCl electrodes, one placed below the lower eyelid and one laterally to the outer canthus of the right eye. Electrode impedances were kept below 10 kΩ.

EEG preprocessing was conducted using EEGLAB [90] and custom-written MATLAB scripts. Continuous EEG data were notch filtered at 50 Hz and harmonics to remove power line noise and then segmented into epochs ranging from −10 to 45 seconds relative to the onset of the sinusoidal stimulation. Differences in EEG baseline across trials were removed by demeaning each trial. EEG data were re-referenced to the common average. Signals contaminated by eye blinks, eye movements, or muscle activities were corrected using independent component analysis [91]. Trials containing excessive signal fluctuations in at least one electrode (amplitude exceeding ±500 μV) were excluded from further analyses. These trials constituted 4.6% of the total number of trials. The corresponding trials in the rating data were also excluded.

Both frequency and phase measures of entrained neural oscillations can be confounded by transient “evoked-type” responses that repeat at the stimulus frequency [6,7,46,48–51]. We did observe a transient response (lasting approximately 0.5 to 2 seconds) locked to the increase of auditory stimulation intensity (S7 Fig). Although interesting, this response could contaminate our measures of ultralow-frequency neural oscillations at the stimulus frequency. We therefore applied a cascade of filters at specifically defined scales in the time domain to both pain and auditory trials, to minimize the potential confounding effects of such regularly occurring transient responses. We first denoised single-trial data after the above processing steps (*s0*) using a moving mean filter with a 0.5-second window (*s1*). We then applied a 2-second median filter and a Gaussian filter with full width at half maximum (FWHM) of 1 second to *s1*, yielding a signal (*s2*) in which the transient responses were removed, whereas the long-period signals were kept. Finally, we reconstructed the EEG signal (*s’*, without the transient responses) as *s’* = *s0 –s1* + *s2*. This algorithm was effective in removing the transient responses while leaving other features of the signal largely intact (S7 Fig). Thus, the power increase and phase locking of the EEG responses revealed by the following analyses were most likely due to a true 0.1-Hz oscillation rather than transient responses that repeated at this frequency. It is also worth noting that although the above-mentioned filters are acausal, their length was too short to affect prestimulus analyses of 0.1-Hz oscillations (i.e., those analyses whose results are shown in Fig 3D).

### Power analysis of EEG data

A fast Fourier transform was applied to single-trial signals ranging from 0 to 30 seconds after the onset of the sinusoidal stimulation, yielding power spectra with a frequency resolution of 0.0333 Hz (a frequency resolution of 0.01 Hz, i.e., spectral interpolation, was achieved by zero padding in the time domain and was used for illustrative purposes). Power estimates were log-transformed and averaged across trials for each participant and condition. To reveal the frequency of power increases, for each participant, condition, electrode, and frequency point, the contribution of background activities (e.g., spontaneous brain activities or slow eye movements) was removed by subtracting the average power at surrounding frequencies (−0.0333 Hz and +0.0333 Hz) [20,36,37]. Scalp topographies of this BSP were computed by spline interpolation.

To identify the frequencies at which power increase occurred, a one-sample one-tailed *t* test was performed at each frequency point to test whether the BSP was consistently greater than zero across participants (FDR corrected for multiple comparisons across frequencies). This analysis was first performed on signals from a central electrode cluster (Cz and its closest neighbors FCC1h, FCC2h, CCP1h, and CCP2h) and then extended to all electrodes (FDR corrected for multiple comparisons across frequencies and electrodes). An additional one-sample one-tailed *t* test was performed separately for each participant and condition, to examine whether the BSP at 0.1 Hz in the central electrode cluster was consistently greater than zero across single trials.

To test the effects of modality and rating task on the power increase detected at 0.1 Hz, we conducted, for each electrode, a two-way repeated-measures ANOVA with factors Modality (two levels: high pain and sound) and Rating (two levels: rating and no rating) on 0.1-Hz BSP (FDR corrected for multiple comparisons across electrodes). Post hoc paired-sample two-tailed *t* tests were performed when a significant main effect or interaction was found.

### Phase analysis of EEG data

We examined the phase locking of the EEG signal (0–30 seconds) across trials by calculating the ITPC [92] for each participant, condition, and electrode. Briefly, given the Fourier phase *φ*_*n*_ for trial *n*, we define the mean vector of phase angles across trials as $m=N^{-1}\sum_{n=1}^{N}e^{i\varphi_{n}}$, where *N* is the number of trials. The ITPC value is given by the modulus of m, i.e., ITPC = |m|. To determine at which frequencies phase locking occurred, we evaluated the ITPC as a function of frequency by calculating the ITPC values in steps of 0.0333 Hz (a frequency resolution of 0.01 Hz was achieved as described in the previous section). ITPC scalp topographies were computed by spline interpolation. For each participant, the significance of ITPC was determined using the Rayleigh’s test for circular uniformity (*P* < 0.05) [39]. The percentage of participants with significant ITPC was calculated for each frequency point, electrode, and condition.

To further assess the significance of the ITPC at the group level (i.e., whether ITPC was greater than what one would expect by chance), the mean ITPC across participants and the percentage of participants with significant ITPC were compared to randomized data. Specifically, we added random phase values drawn from circular uniform distribution to the single trial phases, recalculated the ITPC for each participant, and determined its significance using the Rayleigh’s test. We then computed the mean ITPC across participants and the percentage of participants with significant ITPC. This process was repeated 1,000 times, yielding null distributions of the mean ITPC and of the percentage of participants with significant ITPC. *P* values of the actual data were determined by comparing the mean ITPC and percentage of participants to the respective null distributions. This analysis was also first conducted for the 0.1-Hz oscillation in the central electrode cluster and then extended to other frequencies and electrodes (FDR corrected for multiple comparisons across frequencies and electrodes).

To test the effects of modality and rating on the ITPC at 0.1 Hz, for each electrode, we performed a two-way repeated-measures ANOVA with factors Modality (two levels: high pain and sound) and Rating (two levels: rating and no rating) (FDR corrected for multiple comparisons across electrodes). Post hoc paired-sample two-tailed *t* tests were performed when a significant main effect or interaction was found.

To examine the dependence of the degree of phase locking on the phase of oscillations occurring before stimulus onset, we applied a causal, linear-phase bandpass FIR filter with cutoff frequencies at 0.05 and 0.15 Hz to single-trial EEG signals from the central electrode cluster and then extracted the instantaneous phase of the filtered signals using the Hilbert transform. The causal filter was used to avoid the influence of signals after stimulus onset on the prestimulus phase. We sorted the single trials into six bins from 0 to 2π according to the instantaneous phase at time 0 in the filtered signal. Within each participant, we pooled rating and no-rating trials from the same modality, to increase the number of trials in each bin. We then calculated 0.1-Hz ITPC during the sinusoidal stimulation (0–30 seconds) using the phase obtained from the Fourier transform for the trials within each bin, separately for each participant and modality. Since the number of trials influences ITPC (i.e., fewer trials are more likely to have a greater ITPC value) [93], to correct for differences in the number of trials between bins and modalities, we transformed the ITPC to ITPC_z_ (Rayleigh’s Z) according to the formula *ITPC_z_ = N∙ITPC*^2^, where *N* is the number of trials for each ITPC calculation, as previously recommended [94–96]. Since the causal filter introduced a 5-second delay in the filtered signal (i.e., one-half cycle of a 0.1-Hz oscillation), to represent the relationship for the phase at time 0 in the original ultralow-frequency oscillation without time delay, we added π rad (i.e., one-half cycle) to the phase at time 0 in the filtered signal. Given that the same value was added to all trials, this procedure did not have any effect on the following statistical analyses; importantly, this procedure did not shift the filtered signal and did not introduce noncausality. We performed a two-way repeated-measures ANOVA on the ITPC_z_ with factors Bin (six levels: equal-sized bins from 0 to 2π) and Modality (two levels: pain and sound). Post hoc paired-sample two-tailed *t* tests were performed when a significant main effect or interaction was found. Finally, in addition to the ITPC analyses based on the Fourier phase, we also calculated ITPC using the above-mentioned instantaneous phase to show how ITPC fluctuates over time.

### Analysis of relationship between the strength of neural entrainment and perceived pain intensity across individuals

For each condition entailing a rating task, we calculated across-participant Pearson correlation coefficients between the intensity rating at each time point in the rating time series and the 0.1-Hz BSP as well as the 0.1-Hz ITPC in the central electrode cluster, yielding time series of the correlation coefficient *r* and the *P* value (FDR corrected for multiple comparisons across time points). To test whether the correlations were consequent to the rating task, we performed the same correlation analyses between the intensity ratings and the BSP/ITPC measured in the conditions without rating task. Finally, the same correlation analyses were also performed between conditions entailing different intensities of nociceptive stimulation.

For each participant and condition, we calculated the phase of the entrained 0.1-Hz oscillations as the orientation of the above-defined mean vector m (i.e., arg[m]). To evaluate the across-participant relationship between the intensity rating and the phase of entrained oscillations, we fitted a single-cycle cosine to the participant-mean peak rating (i.e., the peak rating averaged across the three cycles) as a function of the phase of 0.1-Hz oscillations in the central electrode cluster. Significance of the cosine fit was estimated with permutation testing: we randomly permuted the phase values across participants, fitted a cosine function, and calculated the coefficient of determination *R*^2^ as a measure of the goodness of fit. This procedure was repeated 1,000 times, yielding null distribution of the *R*^2^. The *P* value of *R*^2^ obtained from the actual data was determined by comparing it to the null distribution. This analysis was performed between intensity rating and 0.1-Hz phase within each condition entailing the rating task, between intensity rating and 0.1-Hz phase in the conditions without rating task, and between conditions entailing different intensities of nociceptive stimulation.

To ensure that the results from the above analyses were not due to individual variability in stimulus temperature, we performed similar analyses but using stimulus temperature instead of ratings, and also analyzed the correlation between stimulus temperature and pain ratings. Thus, we analyzed across-participant relationships between the laser stimulation temperature and (1) mean peak pain rating (i.e., the peak rating averaged across the three cycles), (2) the BSP, (3) the ITPC, and (4) the phase of 0.1-Hz oscillations in the central electrode cluster.

We further investigated the contribution of the 0.1-Hz BSP, ITPC, and phase difference in explaining the rating variance across participants. We first calculated the correlation between any two of the three explanatory variables (absolute value of the phase difference between the 0.1-Hz oscillation and the stimulus |ΔPhase| is used here), for each of the three pain conditions. We found evidence for correlation in all pairs except in the relationship between BSP and |ΔPhase| in the low-pain rating condition, and a suggestion of a correlation between ITPC and |ΔPhase| in the high-pain no-rating condition (S2 Table). Since the correlations were not too high (correlation coefficients are <0.8, a prerequisite for performing multiple linear regression [97]), we performed multiple linear regression and computed adjusted *R*^2^ and the AIC [41] for regression models containing different combinations of the explanatory variables. These analyses were performed for each pain condition and separately for the ratings of the high-pain and low-pain stimulation as the dependent variable. The adjusted *R*^2^ provides information on the explanatory power of each model. The AIC was used to compare the model fit performance. Specifically, we calculated the difference between the AIC of each model and the minimum AIC in all models (ΔAIC). Based on ΔAIC, we further calculated the Akaike weights *w*_*i*_, as $w_{i}=\frac{exp(-{\Delta AIC}_{i}/2)}{\sum_{m=1}^{M}exp(-{\Delta AIC}_{m}/2)}$, where M is the number of models to be compared, and i is the index of a given model. We finally used these *w*_*i*_ weights as a measure of strength of the evidence in favor of the respective model [42].

### Analysis of neural oscillations outlasting the stimulus

The EEG time series from each individual was denoised using a moving mean filter with a 2-second window and linearly detrended. It should be noted that (1) no zero padding was used at the signal edges, and (2) because of its length, this 2-second moving mean filter, though acausal, cannot produce an oscillation lasting for at least 10 seconds after stimulus offset (Fig 6). Therefore, our observation that the brain oscillations outlasted the stimulus cannot be explained by the temporal smoothing. A one-sample two-tailed *t* test of EEG amplitude against zero was performed at each point of the time series (FDR corrected for multiple comparisons across time points). Scalp topographies of the *t* value were computed over a 1-second window centered around the peak and trough of each cycle. Finally, Pearson correlation coefficients were calculated across participants between the mean peak rating and the peak-to-trough amplitude of the cycle occurring after the sinusoidal stimulation in the central electrode cluster.

### Source modeling of the stimulus-induced oscillations

We estimated the brain generators of ultralow-frequency oscillations using CLARA [43–45], a distributed source analysis approach. Specifically, the scalp topography of the amplitude of each of the three peaks of the stimulus-induced oscillations was extracted from group-mean waveforms, averaged across the three cycles, and imported into the Brain Electrical Source Analysis software (BESA Research version 5.3; MEGIS Software GmbH, Gräfelfing, Germany). A voxel size of 7 mm in Talairach space, singular value decomposition regularization with a cutoff of 0.15%, and three iterations were used to perform the CLARA source analysis [43]. The same analysis was also performed on the scalp topography of the mean amplitude of the three troughs of the oscillations. The source-space results from the mean amplitude of the peaks and the mean amplitude of the troughs were finally averaged. This procedure was performed separately for the three conditions showing a clear entrainment (high-pain no rating, high-pain rating, and low-pain rating).

## Supporting information

S1 Text Supplementary psychophysical results. (DOCX)

S1 Table Across-participants relationship between stimulation temperature and pain ratings, as well as between stimulation temperature and different features of neural entrainment at 0.1 Hz. (DOCX)

S2 Table Across-participants relationships of the 0.1-Hz BSP, ITPC, and phase difference between the entrained oscillation and the stimulus. BSP, background-subtracted power; ITPC, intertrial phase coherence.
(DOCX)

S3 Table Comparison of the seven linear regression models that explain pain rating variance across participants. (DOCX)

S4 Table Order of stimulation trials. (DOCX)

S1 Fig EEG time series from an evenly-spaced set of electrodes (each electrode is in a different column). The signals were smoothed with a moving mean filter with a 2-second window and averaged across participants (*N* = 30). Note the lack of a clear oscillation around 0.1 Hz in the auditory conditions (two bottom rows). Data underlying these plots can be found in S1 Data. EEG, electroencephalography.
(TIF)

S2 Fig Absolute EEG spectra during the rhythmic nociceptive (left) and auditory (right) stimulation in the central electrode cluster. The central electrode cluster included Cz and its closest neighbors FCC1h, FCC2h, CCP1h, and CCP2h. Shaded regions indicate SEM across participants (*N* = 30). The vertical dashed line indicates the frequency of stimulation. Data underlying these plots can be found in S1 Data. EEG, electroencephalography; SEM, standard error of the mean.
(TIF)

S3 Fig Topographies of EEG power enhancement at different frequencies. Topographies of *t* values show strong evidence of EEG power enhancement (expressed as BSP) at 0.1 Hz in central scalp regions, only in the conditions with nociceptive stimulation. Colors indicate scalp electrodes where the BSP had *P* < 0.05 (one-sample *t* test against 0, FDR corrected across electrodes and frequencies). Electrodes with *P* > 0.05 are masked with white. *N* = 30 participants. Data underlying these plots can be found in S1 Data. BSP, background-subtracted power; EEG, electroencephalography; FDR, false discovery rate.
(TIF)

S4 Fig Topographies of EEG phase locking at different frequencies. Topographies showing strong evidence of EEG phase locking at 0.1 Hz in central scalp regions (using two measures: mean ITPC, panel **A**; percentage of participants with significant ITPC, panel **B**), mostly in the conditions with nociceptive stimulation. There was a weak suggestion of phase locking at 0.2 and 0.3 Hz in the auditory condition that also entailed rating. Colors indicate scalp electrodes where the phase locking was greater than chance level (comparison with randomized data; *P* < 0.05, FDR corrected across electrodes and frequencies). Electrodes with *P* > 0.05 are masked with white. *N* = 30 participants. Data underlying these plots can be found in S1 Data. EEG, electroencephalography; ITPC, intertrial phase coherence; FDR, false discovery rate.
(TIF)

S5 Fig ITPC calculated with instantaneous phase from the central cluster of electrodes. Note the building up of phase locking over time. Shaded regions around the solid lines indicate SEM across participants (*N* = 30). Data underlying these plots can be found in S1 Data. ITPC, intertrial phase coherence; SEM, standard error of the mean.
(TIF)

S6 Fig Distributed source analysis of ultralow-frequency oscillations. Sources were estimated using CLARA and superimposed on the BESA standard MRI template. In all three conditions entailing nociceptive stimulation, CLARA estimated two sources: the strongest source was consistently located in the posterior cingulate cortex (left column), whereas the second, weaker source was at the boundary between the insula and putamen (right column). A, anterior; BESA, Brain Electrical Source Analysis; CLARA, classical LORETA analysis recursively applied; Cor, coronal; L, left; P, posterior; R, right; Sag, sagittal; Tra, transverse.
(TIF)

S7 Fig Minimizing the confounding effect of transient responses observed during auditory stimulation with rating. (**A**) EEG signal recorded at electrode Fz (the electrode showing the largest transient response) during auditory stimulation with rating condition, averaged across participants (*N* = 30). Transient responses are visible during each of the three increases of stimulus intensity (blue line, uncorrected signal). The correction algorithm (see Materials and methods) effectively suppressed these transient responses while leaving other features of the signal largely intact (red line, corrected signal). Insets show magnified views of the regions indicated by rectangles. (**B**) Same as (A), but showing signal from a participant with clear transient responses. (**C**) EEG signal recorded at electrode Cz during high pain with rating condition, averaged across participants. Note the lack of the transient responses observed in the auditory condition: the correction algorithm barely affected the recorded signal. (**D**) Same as (C), but showing signal from a single participant. Data underlying these plots can be found in S1 Data. EEG, electroencephalography.
(TIF)

S1 Data Excel spreadsheet containing, in separate sheets, the underlying data for Fig 1, Fig 2, Fig 3, Fig 4, Fig 5, Fig 6, S1 Fig, S2 Fig, S3 Fig, S4 Fig, S5 Fig and S7 Fig. (ZIP)

## Abbreviations

AIC: Akaike information criterion
BSP: background-subtracted power
CLARA: classical LORETA analysis recursively applied
EEG: electroencephalography
ITPC: intertrial phase coherence
PCC: posterior cingulate cortex
SEM: standard error of the mean
VAS: visual analogue scale
