# Supplementary material for: Ultralow-frequency neural entrainment to pain
Source: PLoS Biol. 2020 Apr 13;18(4):e3000491. doi: 10.1371/journal.pbio.3000491 (PMC7179945; doi:10.1371/journal.pbio.3000491)
Supplement: S1 Text — (DOCX) [file pbio.3000491.s001.docx]

**S1 Text. Supplementary psychophysical results.**

For each participant and condition, we extracted the peak amplitude (**Fig 1C, top**) and latency (**Fig 1C, bottom**) of each of the three rating cycles. Two-way repeated-measures ANOVAs with factors Condition (three levels: high pain, low pain, and sound) and Cycle (three levels: 1-3) revealed strong evidence for main effects of the two factors and their interaction (Peak amplitude: Condition, *F*_2,58_=43.09, *P*<0.0001, partial *η*^2^=0.5977; Cycle, *F*_2,58_=86.79, *P*<0.0001, partial *η*^2^=0.7496; interaction, *F*_4,116_=67.62, *P*<0.0001, partial *η*^2^=0.6999. Peak latency: Condition, *F*_2,58_=72.85, *P*<0.0001, partial *η*^2^=0.7153; Cycle, *F*_2,58_=58.61, *P*<0.0001, partial *η*^2^=0.6690; interaction, *F*_4,116_=26.70, *P*<0.0001, partial *η*^2^=0.4793). The peak rating amplitude was higher in the high pain than in the low pain condition (post hoc tests: *P*<0.0001 for each of the three cycles), higher in the auditory than in the low pain condition (*P*<0.0001 for each of the three cycles), as well as higher in the auditory than in the high pain condition, in the last two cycles (both *P*<0.0001) but not in the first cycle (*P*=0.1455). Furthermore, auditory ratings peaked earlier than pain ratings in all three cycles (auditory vs. high pain: *P*=0.0151 [cycle 1], *P*<0.0001 [cycles 2-3]; auditory vs. low pain, *P*<0.0001 [all cycles]), whereas peak latencies in the two pain conditions were not significantly different except for the first cycle, in which high pain latencies were shorter than low pain latencies (cycle 1: *P*=0.0023; cycle 2: *P*=0.7670; cycle 3: *P*=0.0791). Finally, peak amplitudes of pain ratings were smaller and delayed in the last two cycles compared to the first cycle (amplitude: in both high and low pain, cycle 1 vs. cycle 2 or 3, all *P*<0.0001; latency: in both high and low pain, cycle 1 vs. cycle 2 or 3, all *P*<0.0001). This was not the case for the auditory ratings (*P*>0.2009 in all comparisons).
