# Supplementary material for: Ultralow-frequency neural entrainment to pain
Source: PLoS Biol. 2020 Apr 13;18(4):e3000491. doi: 10.1371/journal.pbio.3000491 (PMC7179945; doi:10.1371/journal.pbio.3000491)
Supplement: S1 Table — (DOCX) [file pbio.3000491.s002.docx]

**S1 Table. Across-participants relationship between stimulation temperature and pain ratings, as well as between stimulation temperature and different features of neural entrainment at 0.1 Hz.**

|  | **High Pain, No Rating** | **High Pain, Rating** | **Low Pain, Rating** |
| --- | --- | --- | --- |
| **Pain intensity rating** |  | *r* = -0.2015, *P* = 0.2857 | *r* = -0.1206, *P* = 0.5256 |
| **BSP** | *r* = -0.2142, *P* = 0.2557 | *r* = 0.1955, *P* = 0.3005 | *r* = -0.0715, *P* = 0.7071 |
| **ITPC** | *r* = -0.0472, *P* = 0.8042 | *r* = 0.0962, *P* = 0.6130 | *r* = 0.0020, *P* = 0.9916 |
| **Phase** | *ρ_cl_* = 0.2577, *P* = 0.3695 | *ρ_cl_* = 0.1566, *P* = 0.6922 | *ρ_cl_* = 0.0466, *P* = 0.9679 |

BSP: background-subtracted power. ITPC: intertrial phase coherence. Pain ratings were the peak amplitude averaged across three cycles, in each participant. The 0.1-Hz BSP, ITPC, and phase were measured from the central electrode cluster. For pain ratings, BSP and ITPC, the relationships are expressed as Pearson’s correlation. For phase, the relationship is expressed as circular-linear correlation [1]. N=30 participants.

1. Berens P. CircStat: a MATLAB toolbox for circular statistics. J Stat Softw. 2009;31(10):1-21.
