# Supplementary material for: Ultralow-frequency neural entrainment to pain
Source: PLoS Biol. 2020 Apr 13;18(4):e3000491. doi: 10.1371/journal.pbio.3000491 (PMC7179945; doi:10.1371/journal.pbio.3000491)
Supplement: S2 Table — BSP, background-subtracted power; ITPC, intertrial phase coherence. (DOCX) [file pbio.3000491.s003.docx]

**S2 Table. Across-participants relationships of the 0.1-Hz BSP, ITPC, and phase difference between the entrained oscillation and the stimulus. BSP, background-subtracted power; ITPC, intertrial phase coherence.**

|  | BSP vs. ITPC | BSP vs. \|ΔPhase\| | ITPC vs. \|ΔPhase\| |
| --- | --- | --- | --- |
| High Pain, No Rating | *r* = 0.4718, *P* = 0.0085 | *r* = -0.5630, *P* = 0.0012 | *r* = -0.3557, *P* = 0.0537 |
| High Pain, Rating | *r* = 0.6675, *P* < 0.0001 | *r* = -0.6622, *P* < 0.0001 | *r* = -0.5834, *P* = 0.0007 |
| Low Pain, Rating | *r* = 0.5053, *P* = 0.0044 | *r* = -0.2504, *P* = 0.1821 | *r* = -0.5749, *P* = 0.0009 |

|ΔPhase| denotes absolute value of the phase difference. The 0.1-Hz BSP, ITPC, and |ΔPhase| were measured from the central electrode cluster. The relationships are expressed as Pearson’s correlation. N=30 participants.
