# Supplementary material for: Ultralow-frequency neural entrainment to pain
Source: PLoS Biol. 2020 Apr 13;18(4):e3000491. doi: 10.1371/journal.pbio.3000491 (PMC7179945; doi:10.1371/journal.pbio.3000491)
Supplement: S3 Table — (DOCX) [file pbio.3000491.s004.docx]

**S3 Table. Comparison of the seven linear regression models that explain pain rating** **variance across participants.**

| Model | | DV: High pain rating | | |  | DV: Low pain rating | | |
| --- | --- | --- | --- | --- | --- | --- | --- | --- |
|  |  | *adj. R^2^* | *ΔAIC* | *w_i_* |  | *adj. R^2^* | *ΔAIC* | *w_i_* |
| Condition: High Pain No Rating | BSP | 0.3881 | 4.37 | 0.0517 |  | 0.4429 | 2.11 | 0.1127 |
|  | ITPC | 0.3715 | 5.17 | 0.0346 |  | 0.2752 | 10.01 | 0.0022 |
|  | \|ΔPhase\| | 0.2736 | 9.52 | 0.0039 |  | 0.3741 | 5.61 | 0.0196 |
|  | BSP, ITPC | **0.4804** | **0** | **0.4596** |  | 0.4630 | 1.55 | 0.1491 |
|  | BSP, \|ΔPhase\| | 0.3894 | 4.84 | 0.0409 |  | **0.4900** | **0** | **0.3237** |
|  | ITPC, \|ΔPhase\| | 0.4403 | 2.23 | 0.1507 |  | 0.4437 | 2.61 | 0.0878 |
|  | All three IVs | 0.4910 | 1.15 | 0.2586 |  | 0.5172 | 0.12 | 0.3049 |
|  | | | | | | | | |
| Condition: High Pain Rating | BSP | 0.3509 | 7.31 | 0.0092 |  | 0.3527 | 0.54 | 0.1936 |
|  | ITPC | **0.4913** | **0** | **0.3559** |  | 0.3449 | 0.90 | 0.1617 |
|  | \|ΔPhase\| | 0.3242 | 8.52 | 0.0050 |  | 0.2808 | 3.70 | 0.0399 |
|  | BSP, ITPC | 0.4837 | 0.98 | 0.2180 |  | **0.3753** | **0** | **0.2536** |
|  | BSP, \|ΔPhase\| | 0.3629 | 7.28 | 0.0093 |  | 0.3404 | 1.63 | 0.1123 |
|  | ITPC, \|ΔPhase\| | 0.4956 | 0.28 | 0.3094 |  | 0.3535 | 1.03 | 0.1515 |
|  | All three IVs | 0.4847 | 2.68 | 0.0932 |  | 0.3677 | 2.13 | 0.0874 |
|  | | | | | | | | |
| Condition: Low Pain Rating | BSP | 0.3680 | 7.96 | 0.0081 |  | 0.4336 | 7.13 | 0.0149 |
|  | ITPC | 0.4643 | 3.00 | 0.0963 |  | 0.3972 | 9.00 | 0.0059 |
|  | \|ΔPhase\| | 0.2722 | 12.19 | 0.0010 |  | 0.3020 | 13.40 | 0.0006 |
|  | BSP, ITPC | **0.5238** | **0** | **0.4315** |  | 0.5194 | 2.74 | 0.1341 |
|  | BSP, \|ΔPhase\| | 0.4780 | 2.76 | 0.1085 |  | **0.5613** | **0** | **0.5278** |
|  | ITPC, \|ΔPhase\| | 0.4517 | 4.23 | 0.0520 |  | 0.4088 | 8.95 | 0.0060 |
|  | All three IVs | 0.5403 | 0.71 | 0.3026 |  | 0.5715 | 1.06 | 0.3107 |

BSP: background-subtracted power. ITPC: intertrial phase coherence. |ΔPhase|: absolute value of the phase difference between the 0.1-Hz oscillation and the stimulus. The 0.1-Hz BSP, ITPC, and |ΔPhase| were measured from the central electrode cluster. Pain ratings (the dependent variable, DV) were the peak values averaged across three cycles in each participant. Models with the minimum AIC (i.e., ΔAIC = 0) are shown in bold. N=30 participants.
