# Supplementary material for: Ultralow-frequency neural entrainment to pain
Source: PLoS Biol. 2020 Apr 13;18(4):e3000491. doi: 10.1371/journal.pbio.3000491 (PMC7179945; doi:10.1371/journal.pbio.3000491)
Supplement: S4 Table — (DOCX) [file pbio.3000491.s005.docx]

**S4 Table. Order of stimulation trials.**

| Trials  1-15 | HP  R | AUD  R | LP  R | HP  nR | AUD  R | LP  R | HP  R | AUD  nR | LP  R | HP  nR | AUD  R | LP  R | HP  R | AUD  nR | LP  R |
| --- | --- | --- | --- | --- | --- | --- | --- | --- | --- | --- | --- | --- | --- | --- | --- |
|  |  |  |  |  |  |  |  |  |  |  |  |  |  |  |  |
| Trials  16-30 | **HP**  **nR** | **AUD**  **R** | **LP**  **R** | **HP**  **R** | **AUD**  **nR** | **LP**  **R** | **HP**  **nR** | **AUD**  **R** | **LP**  **R** | **HP**  **R** | **AUD**  **nR** | **LP**  **R** | **HP**  **nR** | **AUD**  **R** | **LP**  **R** |
|  |  |  |  |  |  |  |  |  |  |  |  |  |  |  |  |
| Trials  31-45 | **HP**  **R** | **AUD**  **nR** | **LP**  **R** | **HP**  **nR** | **AUD**  **R** | **LP**  **R** | **HP**  **R** | **AUD**  **nR** | **LP**  **R** | **HP**  **nR** | **AUD**  **R** | **LP**  **R** | **HP**  **R** | **AUD**  **nR** | **LP**  **R** |
|  |  |  |  |  |  |  |  |  |  |  |  |  |  |  |  |
| Trials  46-60 | **HP**  **nR** | **AUD**  **R** | **HP**  **R** | **AUD**  **nR** | **HP**  **nR** | **AUD**  **R** | **HP**  **R** | **AUD**  **nR** | **HP**  **nR** | **AUD**  **R** | **HP**  **R** | **AUD**  **nR** | **HP**  **nR** | **AUD**  **R** | **HP**  **R** |
|  |  |  |  |  |  |  |  |  |  |  |  |  |  |  |  |
| Trials  61-75 | **AUD**  **nR** | **HP**  **nR** | **AUD**  **R** | **HP**  **R** | **AUD**  **nR** | **HP**  **nR** | **AUD**  **R** | **HP**  **R** | **AUD**  **nR** | **HP**  **nR** | **AUD**  **R** | **HP**  **R** | **AUD**  **nR** | **HP**  **nR** | **AUD**  **nR** |

HP: high-pain stimulation. LP: low-pain stimulation. AUD: auditory stimulation. R: trials in which participants were required to continuously rate perceived intensity. nR: trials without the rating task. Participants were allowed to rest for approximately 2 minutes after every 15 trials.
