# Supplementary figures and images for: Ultralow-frequency neural entrainment to pain
Source: PLoS Biol. 2020 Apr 13;18(4):e3000491. doi: 10.1371/journal.pbio.3000491 (PMC7179945; doi:10.1371/journal.pbio.3000491)

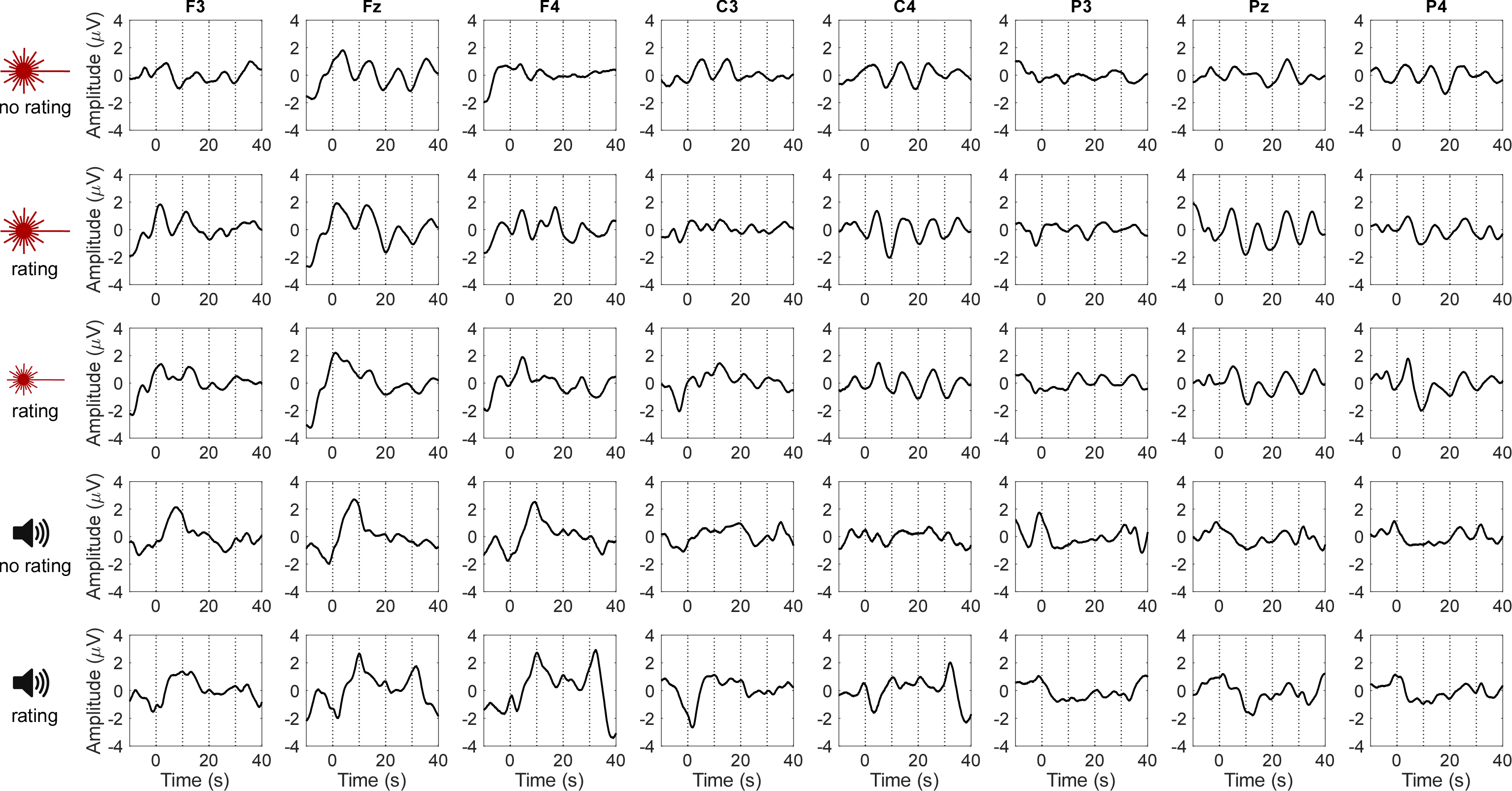

Supplement: S1 Fig — The signals were smoothed with a moving mean filter with a 2-second window and averaged across participants (N = 30). Note the lack of a clear oscillation around 0.1 Hz in the auditory conditions (two bottom rows). Data underlying these plots can be found in S1 Data. EEG, electroencephalography. (TIF) [file pbio.3000491.s006.tif]

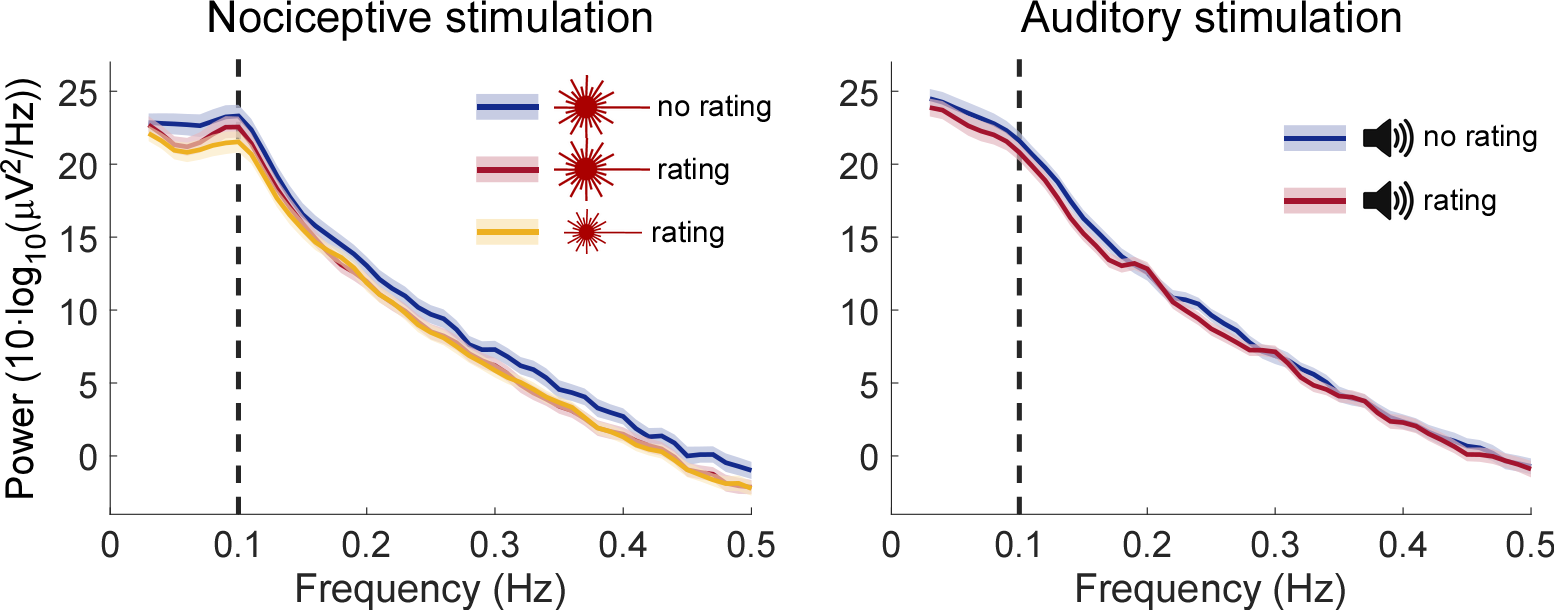

Supplement: S2 Fig — The central electrode cluster included Cz and its closest neighbors FCC1h, FCC2h, CCP1h, and CCP2h. Shaded regions indicate SEM across participants (N = 30). The vertical dashed line indicates the frequency of stimulation. Data underlying these plots can be found in S1 Data. EEG, electroencephalography; SEM, standard error of the mean. (TIF) [file pbio.3000491.s007.tif]

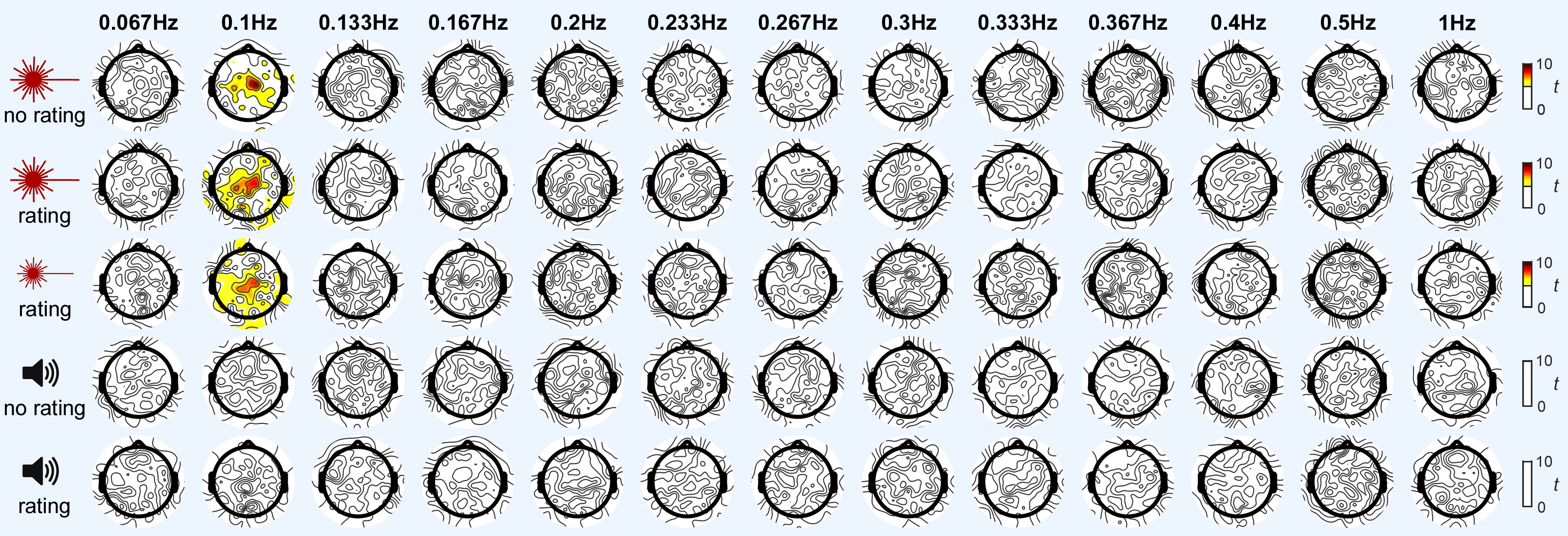

Supplement: S3 Fig — Topographies of t values show strong evidence of EEG power enhancement (expressed as BSP) at 0.1 Hz in central scalp regions, only in the conditions with nociceptive stimulation. Colors indicate scalp electrodes where the BSP had P < 0.05 (one-sample t test against 0, FDR corrected across electrodes and frequencies). Electrodes with P > 0.05 are masked with white. N = 30 participants. Data underlying these plots can be found in S1 Data. BSP, background-subtracted power; EEG, electroencephalography; FDR, false discovery rate. (TIF) [file pbio.3000491.s008.tif]

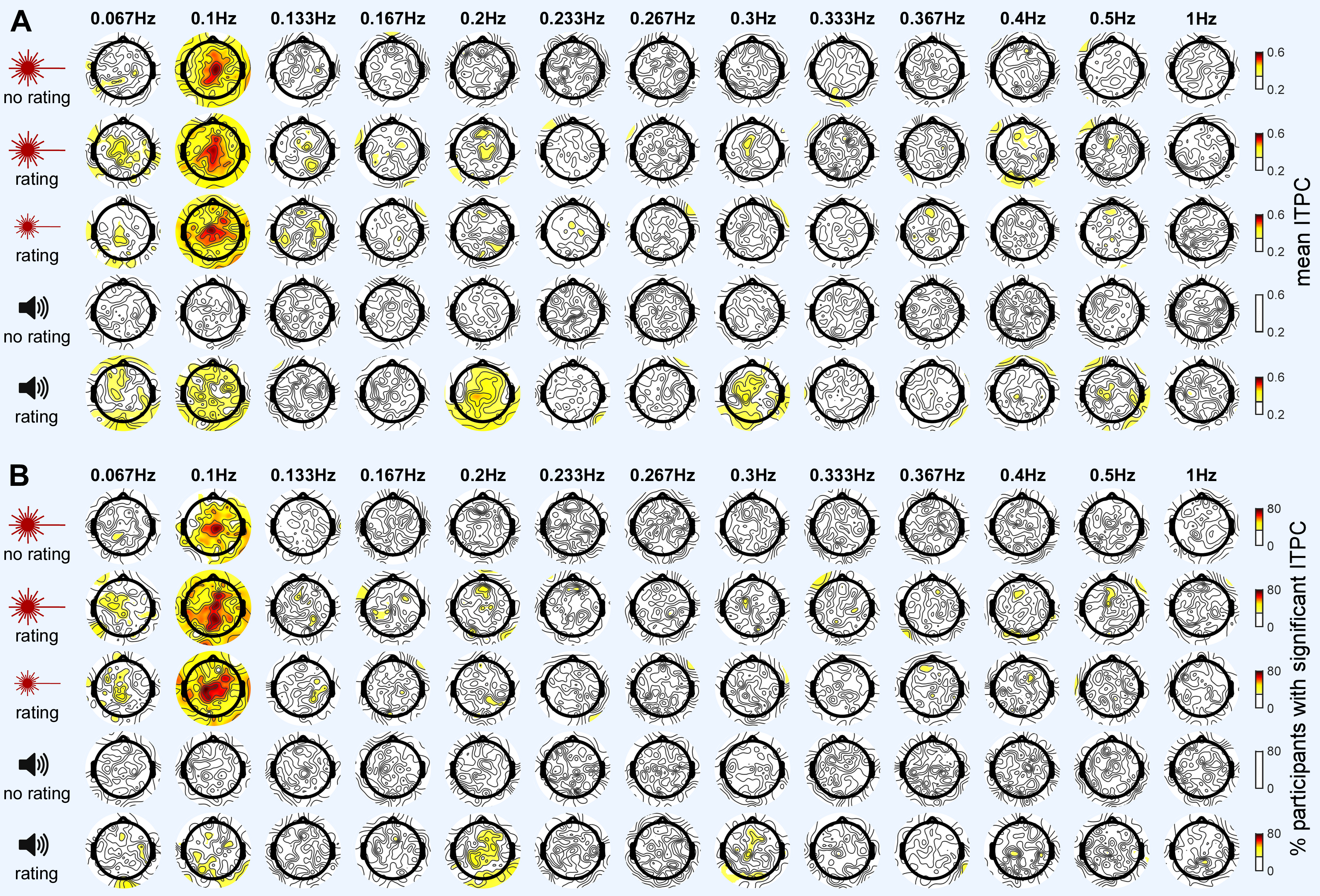

Supplement: S4 Fig — Topographies showing strong evidence of EEG phase locking at 0.1 Hz in central scalp regions (using two measures: mean ITPC, panel A; percentage of participants with significant ITPC, panel B), mostly in the conditions with nociceptive stimulation. There was a weak suggestion of phase locking at 0.2 and 0.3 Hz in the auditory condition that also entailed rating. Colors indicate scalp electrodes where the phase locking was greater than chance level (comparison with randomized data; P < 0.05, FDR corrected across electrodes and frequencies). Electrodes with P > 0.05 are masked with white. N = 30 participants. Data underlying these plots can be found in S1 Data. EEG, electroencephalography; ITPC, intertrial phase coherence; FDR, false discovery rate. (TIF) [file pbio.3000491.s009.tif]

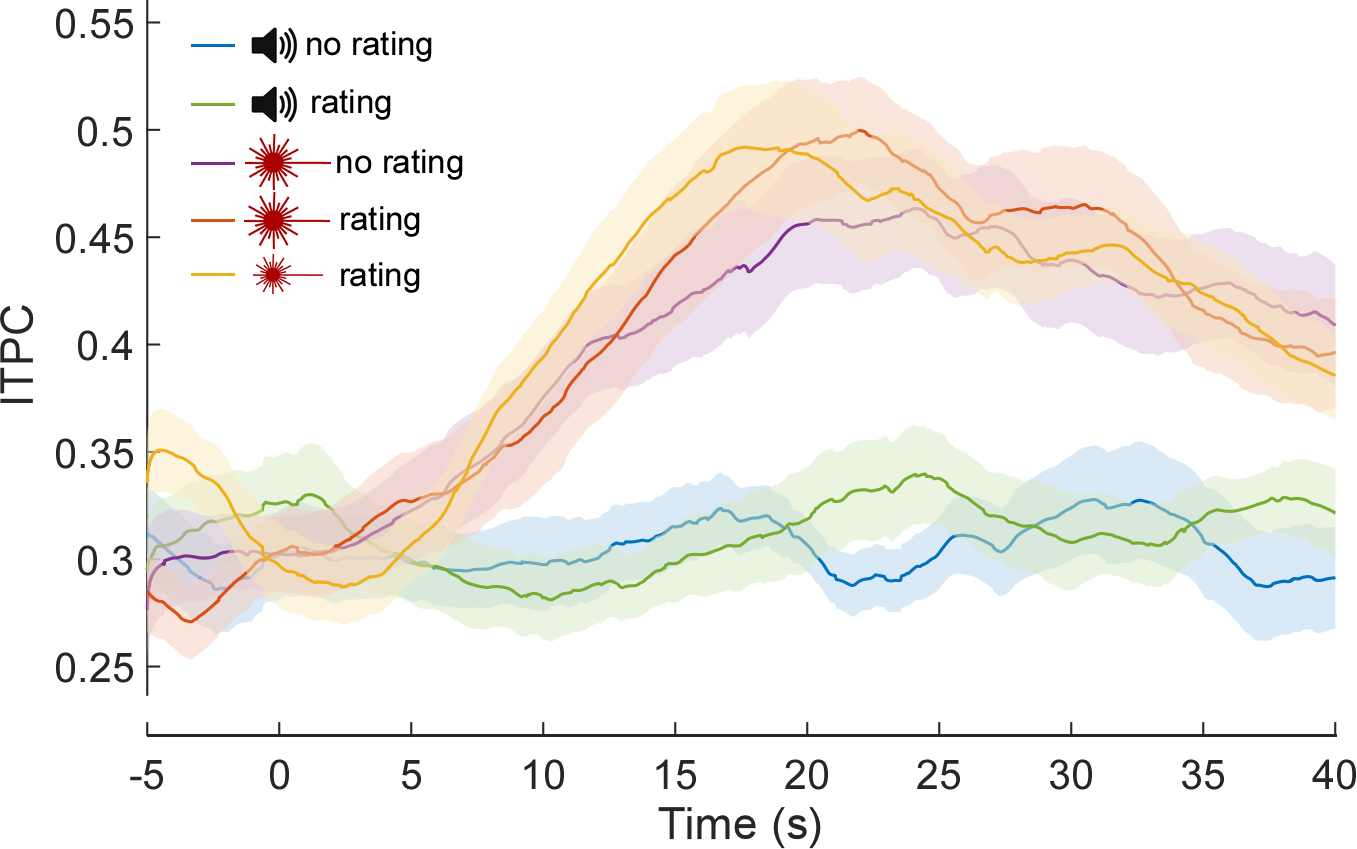

Supplement: S5 Fig — Note the building up of phase locking over time. Shaded regions around the solid lines indicate SEM across participants (N = 30). Data underlying these plots can be found in S1 Data. ITPC, intertrial phase coherence; SEM, standard error of the mean. (TIF) [file pbio.3000491.s010.tif]

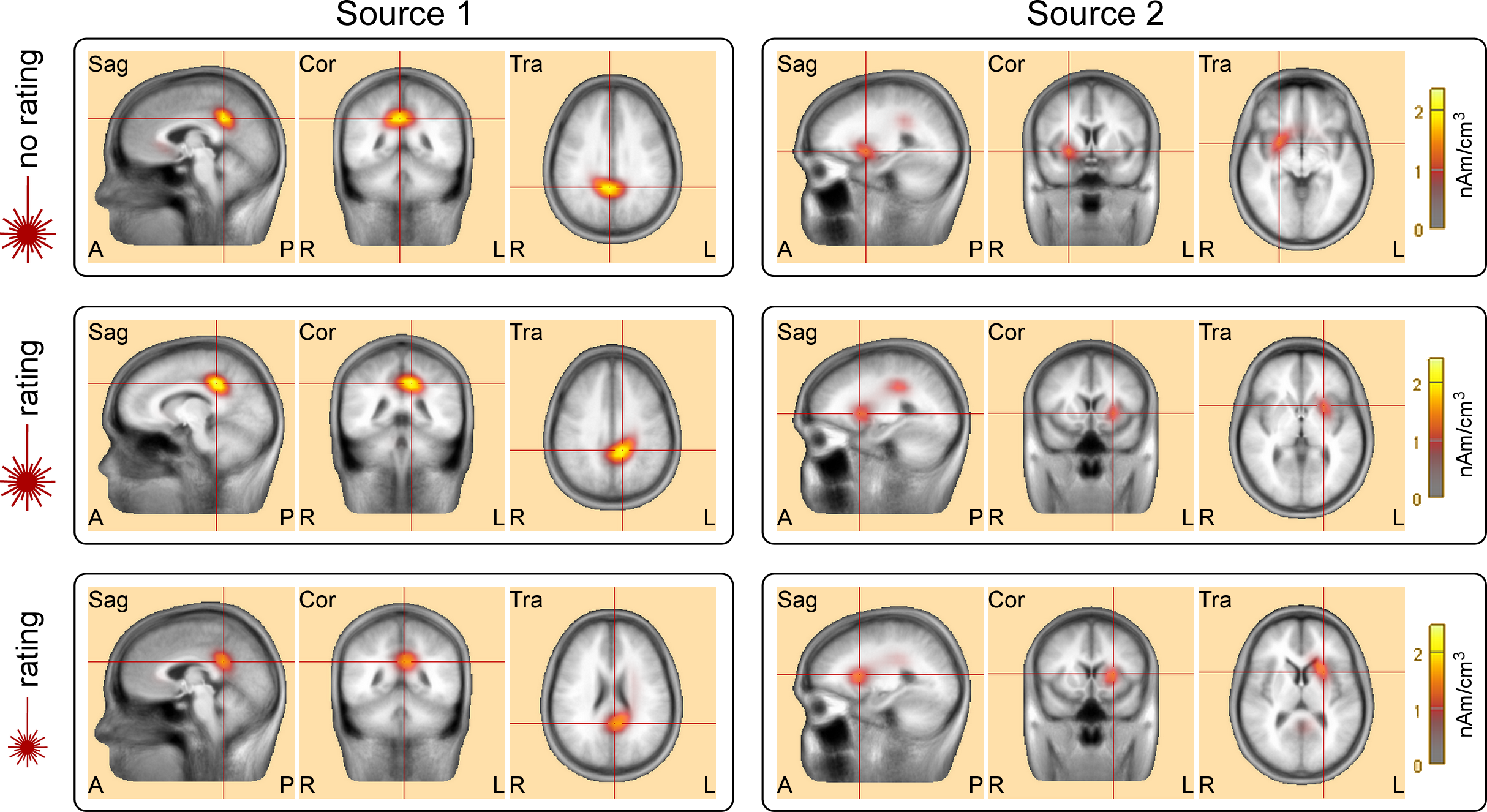

Supplement: S6 Fig — Sources were estimated using CLARA and superimposed on the BESA standard MRI template. In all three conditions entailing nociceptive stimulation, CLARA estimated two sources: the strongest source was consistently located in the posterior cingulate cortex (left column), whereas the second, weaker source was at the boundary between the insula and putamen (right column). A, anterior; BESA, Brain Electrical Source Analysis; CLARA, classical LORETA analysis recursively applied; Cor, coronal; L, left; P, posterior; R, right; Sag, sagittal; Tra, transverse. (TIF) [file pbio.3000491.s011.tif]

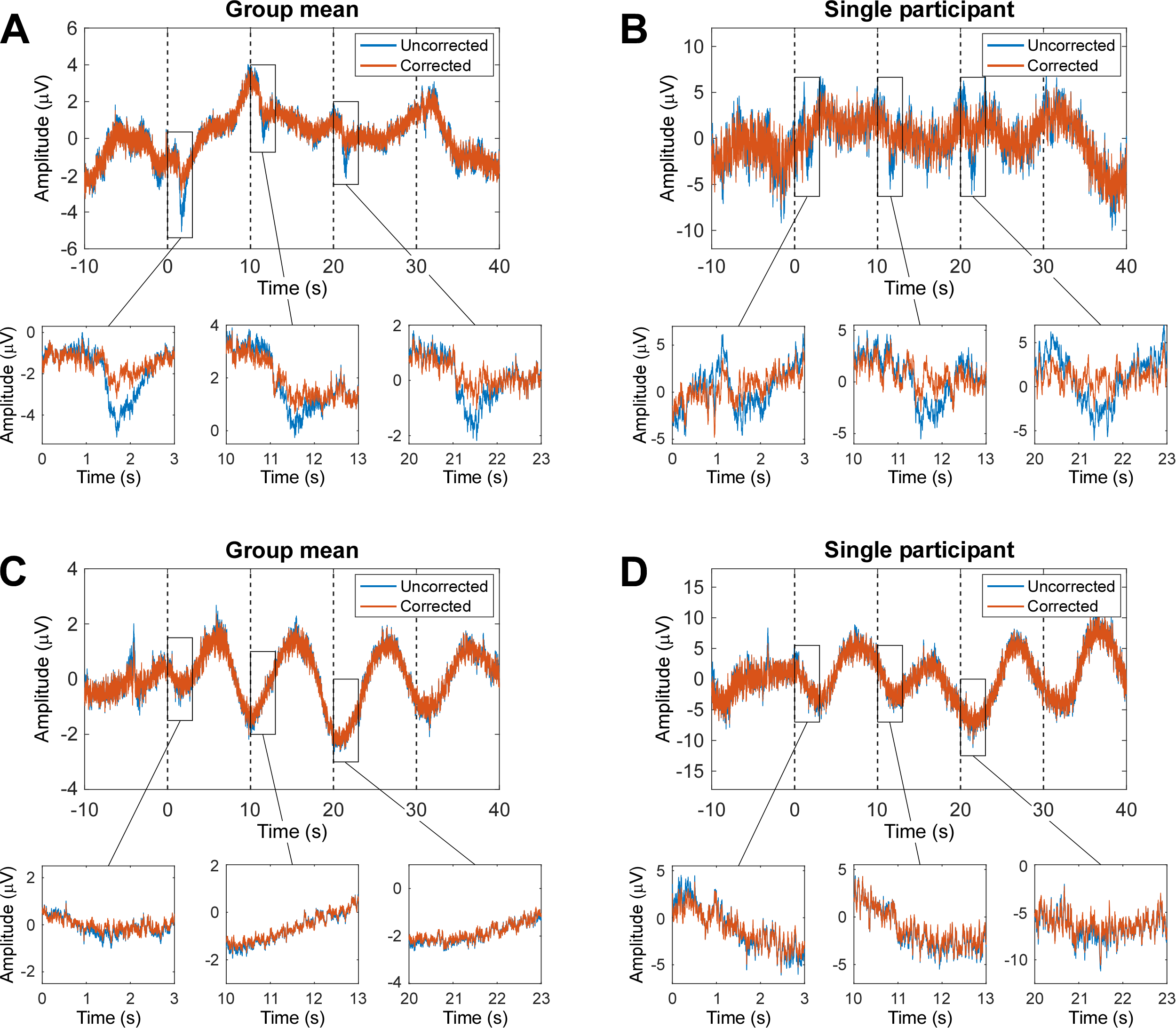

Supplement: S7 Fig — (A) EEG signal recorded at electrode Fz (the electrode showing the largest transient response) during auditory stimulation with rating condition, averaged across participants (N = 30). Transient responses are visible during each of the three increases of stimulus intensity (blue line, uncorrected signal). The correction algorithm (see Materials and methods) effectively suppressed these transient responses while leaving other features of the signal largely intact (red line, corrected signal). Insets show magnified views of the regions indicated by rectangles. (B) Same as (A), but showing signal from a participant with clear transient responses. (C) EEG signal recorded at electrode Cz during high pain with rating condition, averaged across participants. Note the lack of the transient responses observed in the auditory condition: the correction algorithm barely affected the recorded signal. (D) Same as (C), but showing signal from a single participant. Data underlying these plots can be found in S1 Data. EEG, electroencephalography. (TIF) [file pbio.3000491.s012.tif]
